# Alternative oxidase (AOX) constitutes a small family of proteins in *Citrus clementina* and *Citrus sinensis* L. Osb

**DOI:** 10.1371/journal.pone.0176878

**Published:** 2017-05-01

**Authors:** Jacqueline Araújo Castro, Monique Drielle Gomes Ferreira, Raner José Santana Silva, Bruno Silva Andrade, Fabienne Micheli

**Affiliations:** 1 Universidade Estadual de Santa Cruz (UESC), Centro de Biotecnologia e Genética (CBG), Ilhéus, Bahia, Brazil; 2 Instituto Federal de Educação, Ciência e Tecnologia Baiano (IFBaiano), Santa Inês, Bahia, Brazil; 3 Universidade Estadual Sudoeste da Bahia (UESB), Av. José Moreira Sobrinho, Jequié, Bahia, Brazil; 4 CIRAD, UMR AGAP, F-34398 Montpellier, France; Fujian Agriculture and Forestry University, CHINA

## Abstract

The alternative oxidase (AOX) protein is present in plants, fungi, protozoa and some invertebrates. It is involved in the mitochondrial respiratory chain, providing an alternative route for the transport of electrons, leading to the reduction of oxygen to form water. The present study aimed to characterize the family of *AOX* genes in mandarin (*Citrus clementina*) and sweet orange (*Citrus sinensis*) at nucleotide and protein levels, including promoter analysis, phylogenetic analysis and *C*. *sinensis* gene expression. This study also aimed to do the homology modeling of one AOX isoform (CcAOXd). Moreover, the molecular docking of the CcAOXd protein with the ubiquinone (UQ) was performed. Four *AOX* genes were identified in each citrus species. These genes have an open reading frame (ORF) ranging from 852 bp to 1150 bp and a number of exons ranging from 4 to 9. The 1500 bp-upstream region of each *AOX* gene contained regulatory *cis*-elements related to internal and external response factors. *CsAOX* genes showed a differential expression in citrus tissues. All AOX proteins were predicted to be located in mitochondria. They contained the conserved motifs LET, NERMHL, LEEEA and RADE-H as well as several putative post-translational modification sites. The CcAOXd protein was modeled by homology to the AOX of *Trypanosona brucei* (45% of identity). The 3-D structure of CcAOXd showed the presence of two hydrophobic helices that could be involved in the anchoring of the protein in the inner mitochondrial membrane. The active site of the protein is located in a hydrophobic environment deep inside the AOX structure and contains a diiron center. The molecular docking of CcAOXd with UQ showed that the binding site is a recessed pocket formed by the helices and submerged in the membrane. These data are important for future functional studies of citrus AOX genes and/or proteins, as well as for biotechnological approaches leading to AOX inhibition using UQ homologs.

## Introduction

The term oxidase refers to any enzyme that catalyzes the oxidation–reduction reaction involving molecular oxygen as an electron acceptor. In these reactions, the oxygen is reduced to water or to hydrogen peroxide. The alternative oxidase (AOX) protein is present in plants, fungi, protozoa and some invertebrates, but it has not been found in mammals. It is located on the matrix side of the inner mitochondrial membrane and is involved in the mitochondrial respiratory chain, providing an alternative route for the passage of electrons. The main electron transport route in eukaryotes passes through the complex IV (known as cyanide-sensitive cytochrome oxidase) of the respiratory chain, but in some organisms the electron transport route goes through the AOX protein (known as cyanide-insensitive and hydroxamic acid-sensitive terminal oxidase). Both routes lead to the transportation of electrons and the reduction of oxygen to form water [1, 2]. However, the transportation through the AOX protein occurs without the pumping of protons into the intermembrane space and consequently is not coupled with ATP synthesis and energy conservation [3]. The AOX catalyzes the four-electron oxidation of ubiquinol (reduced form of ubiquinone [UQ]) by oxygen, and the energy of ubiquinol oxidation by oxygen is released as heat [3–5].

The AOX proteins (32–36 kDa) are encoded by a family of nuclear genes [6], and several studies report that, in plants, variations of environmental factors such as abiotic stresses, pathogen infection and oxidative stress may influence the expression of *AOX* genes [3, 7–10]. Moreover, AOX has been proposed to play a role in homeostasis and plant growth [11] and in maintaining metabolic flexibility for rapid adaptation to stress [12]. In citrus plants, the only studies of AOX proteins have been related to abiotic stresses (e.g., drought, boron tolerance) [13–15], and no genome-wide characterization of the AOX family has yet been performed for this genus. The availability of the data from the recent sequencing of the genome of some citrus species (https://www.citrusgenomedb.org/) allowed for the genome-wide analysis of gene families as a pre-requisite for functional and/or pre-breeding studies. The present study aimed to characterize the family of *AOX* genes in mandarin (*C*. *clementina*) and sweet orange (*C*. *sinensis*) at nucleotide and protein levels, including promoter analysis. The study also aimed to construct the homology modeling of one AOX isoform (CcAOXd). Moreover, the molecular docking of the CcAOXd protein with the UQ was performed.

## Material and methods

### *In silico* analysis of AOX citrus genes and proteins

The identification and structural analysis of the *AOX* genes (introns/exons) were performed using the Citrus Genome Database (https://www.citrusgenomedb.org/). Open reading frame (ORF) analysis was performed using the ORFinder software (http://www.ncbi.nlm.nih.gov/orffinder/). The prediction of the theoretical isoelectric point (pI) and the molecular weight (MW) was obtained using the pI/Mw tool (www.expasy.org). Conserved domain and family protein were analyzed using the Pfam (http://pfam.sanger.ac.uk/search/sequence) and InterProScan software [16]. The predictions of the subcellular location of the protein and of the location of the cleavage site were performed by the MitoProt II software (https://ihg.gsf.de/ihg/mitoprot.html). Transmembrane helices were predicted using the TMPred software [17], whereas hydropathicity levels were identified using the ProtScale program (http://web.expasy.org/protscale/). The NetPhos 3.1 Server [18] and the NetNGlyc 1.0 Server (http://www.cbs.dtu.dk/services/NetNGlyc/) were used to identify putative phosphorylation sites (Ser/Thr/Tyr) and putative N-glycosylation sites (Asn-X-Ser/Thr type), respectively. The protein motif analysis was conducted using the program MEME/MAST [19]. The maximum number of motifs was set to 20, the maximum motif length was set to 80 amino acids, the optimum motif width was constrained to between 6 and 300 residues, and the other parameters were used as default.

### Analysis of the promoter regions and chromosomal locations of *AOX* genes

To identify the presence of the *cis*-regulatory elements in the promoter regions of the *AOX* genes, the 1500 bp upstream region from the translation start site of the genes was analyzed using the plantCARE (sphinx.rug.ac.be:8080/PlantCARE/cgi/index.html) software [20]. The chromosomal locations of the *AOX* genes were obtained by screening the GFF3 file of each genome (*C*. *clementin*a and *C*. *sinensis* deposited in the Citrus Genome Database) using the AOX sequence ID.

### Phylogeny

Phylogenetic analysis was performed based on the alignment of the amino acid sequence of the AOX proteins from *C*. *sinensis* and *C*. *clementina* with alternative oxidase proteins from *Arabidopsis thaliana*. The sequences were aligned with ClustalW2 (http://www.ebi.ac.uk/Tools/msa/clustalw2/) [21]. The MEGA 5.1 program [22] was used to construct a phylogenetic tree by using the neighbor-joining statistical method [23] reliably established by 1000 bootstrap samples.

### Molecular modeling

To select the best 3-D template for AOX molecular modeling from resolved 3-D structures, the AOX proteins from *C*. *clementina* and *C*. *sinensis* were aligned with the Protein Data Bank (Pdb) using the PSIBLAST program [24]. Target 3-D structures were modeled using templates that presented the highest identity and coverage, starting from a minimum of 25% of identical amino acids in the alignment. Additionally, the minimum template resolution considered was 2.0 Å. The predicted 3-D protein model was obtained using the SWISS-MODEL server (https://swissmodel.expasy.org) and the Swiss-Pdb Viewer program v.3.7 [25]. The α-carbon chain RMSD between targets and their respective templates was calculated using PyMOL V3.0 [26]. The stereochemical quality of both AOX models was calculated by Procheck 3.4 [27] and the Atomic Non-Local Environment Assessment (ANOLEA) program [28]. The validation of the secondary structure was performed using the Protein Structure Prediction Server-PSIPRED program [29].

### Molecular docking of CcAOXd with ubiquinone

Before preforming the docking between the ligand and the target protein, the ubiquinone (UQ) structure (C_59_H_90_O_4_) was downloaded from pubchem database (https://pubchem.ncbi.nlm.nih.gov/) in SMILES format. The UQ structure was converted into 3-D format using MarvinSketch 15.7.13.0 (https://www.chemaxon.com/products/marvin/marvinsketch/) and saved in mol2 format. Furthermore, AutoDockTools V1.5.6 [30] was used to prepare the protein and UQ structure for docking calculations. First, polar hydrogens were added to the UQ structure and all torsions were checked; the ligand structure was then saved in PDBQT format. Based on the alignment between CcAOXd and AOX structures, the amino acids of the active were marked in order to get the grid box coordinates for the docking process. Afterward, the CcAOXd structure was saved in PDBQT format. Calculations for the docking between CcAOXd and UQ were performed using AutoDock Vina software [30] considering 9 different docking poses and based on UQ bond torsions. All docking results were evaluated using PyMOL V1.7.4 [26] in order to check which UQ poses appeared in the CcAOXd active site and to identify which pose presents the best docking affinity energy. Additionally, Discovery Studio 4.5 was used to generate the 2-D map of the interaction between CcAOXd and UQ.

### *In silico C*. *sinensis AOX* gene expression

*CsAOXa*, *CsAOXb*, *CsAOXc* and *CsAOXd* gene sequences were blasted on the *Citrus sinensis* Annotation Project database (CAP; http://citrus.hzau.edu.cn/orange/ [31]) to obtain the CAP accession number of each gene. Using the CAP accession number, the complete data of each gene, including the RNA-seq gene expression values in four tissues (callus, leaf, flower and fruit) was obtained [31].

## Results and discussion

### *AOX* gene family in the sweet orange and tangerine genomes

**Fig 1:**
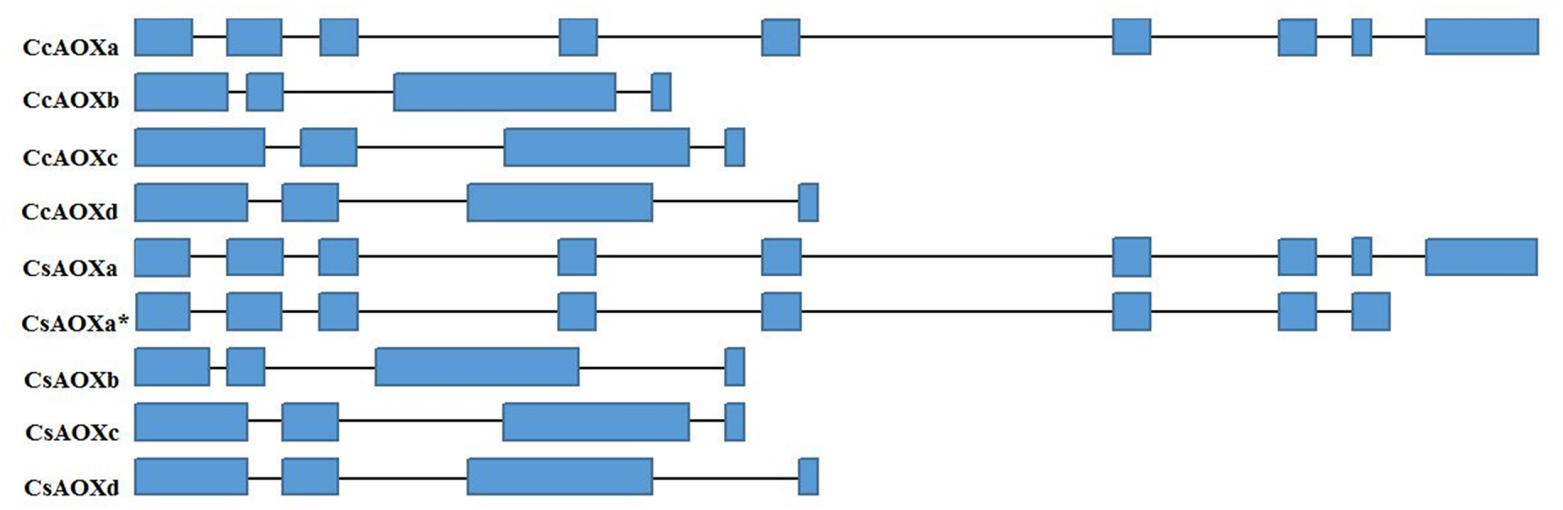
Structure of *AOX* genes from *C*. *clementina* and *C*. *sinensis*. Blue squares represent the exons and black lines represent the introns. (*) indicated the gene ID of the alternative transcript of the *CsAOXa* gene.

Existing annotation in the Citrus Genome Database allowed for the identification of a total of 8 *AOX* genes, with 4 belonging to *C*. *clementina* (named *CcAOXa*, *CcAOXb*, *CcAOXc* and *CcAOXd*) and 4 to *C*. *sinensis* (named *CsAOXa*, *CsAOXb*, *CsAOXc* and *CsAOXd*; Table 1). The *CcAOX* genes were distributed in chromosomes 2, 5 and 8 (Table 1). The gene ORFs ranged from 927 to 1150 bp, and the number of exons ranged from 4 to 9 (Table 1; Fig 1; S1 Fig). The *CsAOX* genes were located in chromosomes 2, 3 and 8 (Table 1). The gene ORFs ranged from 852 to 1050 bp, and the number of exons ranged from 4 to 9 (Table 1; Fig 1; S1 Fig). For most of the genes, the 5’ end of the introns presented the GT sequence as a splicing donation site, whereas the 3’ end presented the AG sequence as a splicing acceptor site (S1 Fig). The number of *AOX* genes found in *C*. *clementina* and *C*. *sinensis* is small, which is similar to what has been observed in other species such as *Arabidopsis thaliana*, whose *AOX* family is represented by five genes [7]; *Glycine max* [32], *Oryza sativa* [33] and *Zea mays* [34], each represented by three genes; and *Nicotiana tabacum* [2], *Triticum aestivum* [35] and *Hypericum perforatum* [36], each represented by two genes. Most of the *AOX* genes in this study have structures with 4 exons and 3 introns, which has also been observed in other species such as *A*. *thaliana*, *G*. *max*, *Theobroma cacao*, *Citrus sinensis*, *Gossypium hirsutum*, *O*. *sativa*, *T*. *aestivum*, *Vigna unguiculata*, *Vitis vinifera* and *Z*. *mays* [36, 37]. In contrast to the 4-exon structure reported for most of the organisms, the *CcAOXa* and *CsAOXa* genes presented 9 exons and 8 introns. Genes that are readily adjustable–for example, those that respond to stress–generally exhibit a smaller number of introns, which results in a slower response time for the production of the protein and gives to these genes them a selective advantage [38]. The presence of introns may result in production delays due to the steps required for splicing and transcription, as well as an additional energy costs caused by the additional length of the nascent transcript [38]. Collinearity analysis was performed for the *AOX* genes in the *C*. *clementina* and *C*. *sinensis* genomes using the MCScanX toolkit, and the analysis showed that the citrus *AOX* genes did not come from duplication events (data not shown).

**Table 1:** Characteristics of the *AOX* genes present in the *Citrus clementina* and *Citrus sinensis* genomes. ORF: open reading frame. (*) indicated the gene ID of the alternative transcript of the *CsAOXa* gene.

Species	Gene name	Gene ID	Location	ORF size (bp)	Quantity of introns	Quantity of exons
*C*. *clementina*	*CcAOXa*	clementine0.9_012574m	Chromosome 2	1150	8	9
	*CcAOXb*	clementine0.9_034013m	Chromosome 5	927	3	4
	*CcAOXc*	clementine0.9_015158m	Chromosome 5	1011	3	4
	*CcAOXd*	clementine0.9_015716m	Chromosome 8	978	3	4
*C*. *sinensis*	*CsAOXa* CsAOXa*	orange1.1g018864m orange1.1g022654m*	Chromosome 2	1050	8 7	9 8
	*CsAOXb*	orange1.1g037339m	Chromosome 3	852	3	4
	*CsAOXc*	orange1.1g019765m	Chromosome 3	1008	3	4
	*CsAOXd*	orange1.1g020532m	Chromosome 8	960	3	4

### Promoter sequence analysis of the citrus *AOX* genes

**Fig 2:**
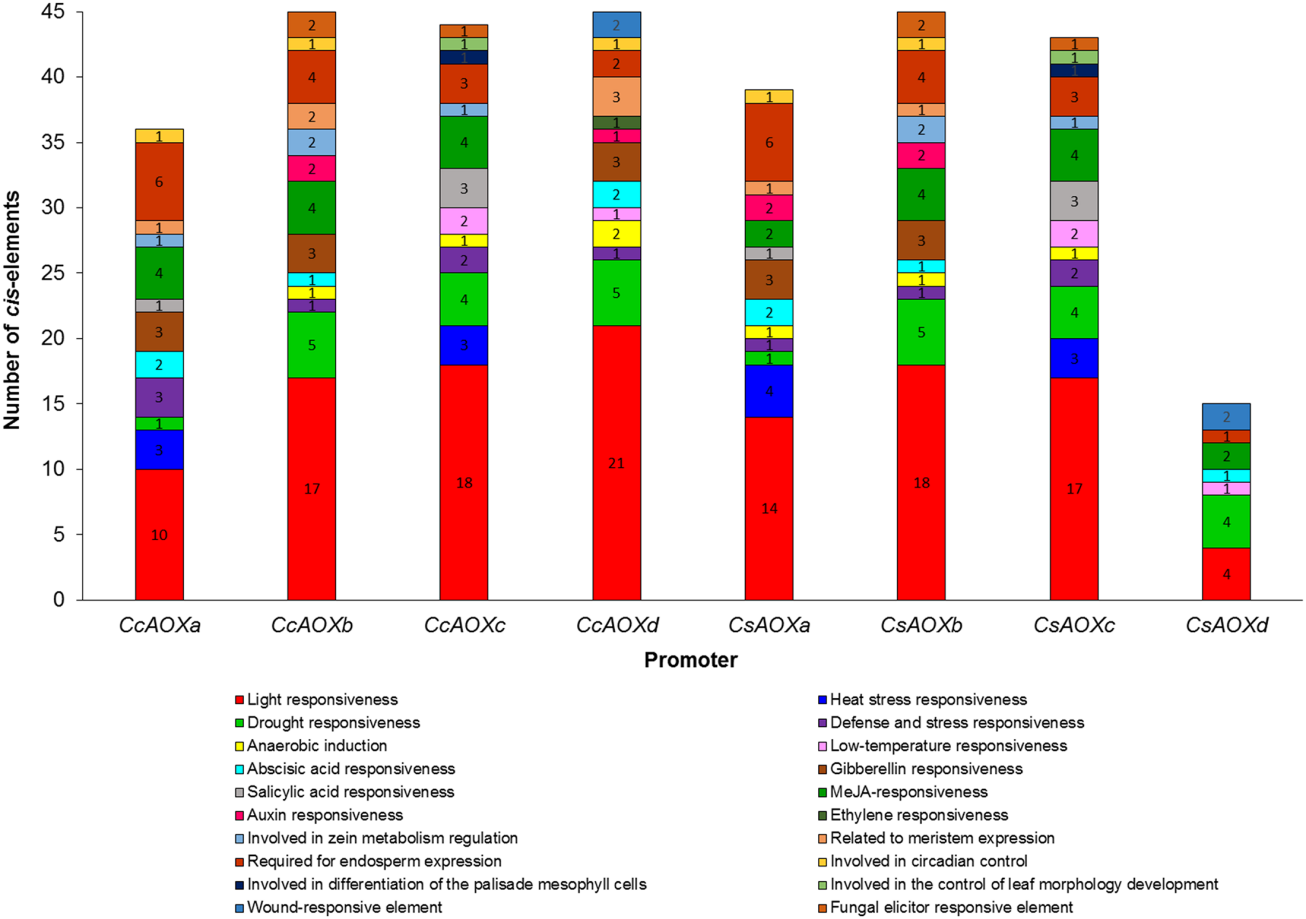
*Cis*-elements present in the promoter region of citrus *AOX* genes. The *cis*-elements were analyzed in the upstream promoter region of the translation start site using the plantCARE database.

A fragment belonging to the upstream region of each *AOX* gene was analyzed to find plant-specific *cis*-elements using the PlantCARE database. Except for the *CsAOXd* gene, for which the only fragment available in the Citrus Genome Database was 353 bp in length, the promoter fragment size used was 1500 bp (S2 Fig). The TATA and CAAT-box elements were found in all citrus AOX promoter regions (S3 Fig); the other *cis*-elements varied between sequence promoters (Fig 2, S3 Fig). Most of the *cis*-elements (quantity of 4 to 21, according to the promoter) were involved in the response to light (Fig 2). In smaller proportions, *cis*-elements were found that were responsive to i) hormones or inducers such as methyl jasmonate (MeJA), gibberellin, ethylene, auxin, abscisic acid and salicylic acid; and ii) biotic, abiotic or mechanical stresses such as drought, wounds, heat, low temperature, fungal elicitors and anaerobiosis. Others *cis*-elements related to plant development such as zein metabolism, endosperm expression, differentiation of palisade mesophyll cells, meristem expression, circadian control and leaf morphology were also present in the promoters of the citrus *AOX* genes (Fig 2). This analysis revealed a large number of motifs responding to different external or endogen inductions, suggesting a complex regulation of *AOX* gene expression. Under stress conditions, it is common to observe the accumulation of reactive oxygen species and/or of molecules or ion such as salicylic acid, jasmonate, calcium and ethylene in the organism [39]. All these signaling molecules have the ability to induce *AOX* gene expression [40–42]. Indeed, the overexpression of *AOX* genes has already been reported in response to a number of biotic and abiotic stresses [5, 43, 44]. In *Arabidopsis thaliana*, the mutants *AOX1a*-deficient and *AOX1b*-deficient were more severely photodamaged by high light intensity when compared with wild-type plants [45]. These results indicated that in high light intensity conditions, *AOX1a* and *AOX1b* genes may favor plant adaptation. According to Feng et al. [8], light may induce *AOX* gene expression by increasing ROS production.

### Analysis of the citrus AOX proteins

The number of amino acid residues of the citrus AOX proteins ranged from 284 (CsAOXb) to 349 (CsAOXa) (Table 2). All proteins were predicted to be located in mitochondria (73.00% to 99.69% probability; Table 2) because they had mitochondrial protein targeting region (S3 Fig). According to MitoProt II, these regions were located in the N-terminal portion of the protein, with the amount of amino acid residue ranging from 30 to 49 (Table 2; S4 Fig). The hydropathicity of the proteins ranged from -0.384 to -0.145 (Table 2). The mitochondrial targeting of the citrus AOX proteins was predicted with a high probability, which suggested that organelle isolations will be required to analyze AOX proteins *in vitro*. Although all AOX proteins showed mitochondrial targeting regions, the alignment of these regions did not allow for any clear prediction or conserved sequence identification. Moreover, a high variability in the nucleotidic N-terminal region was observed across the citrus *AOX* genes (both within and between species; data not shown). It is still not known how this variability can affect the regulation of gene expression and/or the protein transport or activity.

**Table 2:** Characteristics of the AOX proteins present in the citrus genomes. GRAVY: grand average of hydropathicity; Mw: molecular weight; pI: isoeletric point; SP: signal peptide. *Protein resulting from the alternative transcript of the *CsAOXa* gene.

Protein	Protein size (aa)	pI with/without SP	Mw with/without SP (kDa)	Export probability to mitochondria (%)	SP size (aa)	GRAVY
CcAOXa	349	6.09 / 5.26	39.9 / 34.5	99.55	49	-0.210
CcAOXb	309	8.27 / 6.36	35.2 / 30.1	99.47	45	-0.329
CcAOXc	336	8.81 / 6.68	38.1 / 32.8	99.36	49	-0.384
CcAOXd	325	8.29 / 6.68	37.1 / 33.9	73.00	30	-0.183
CsAOXa	349	5.64 / 5.07	40.2 / 34.7	99.65	49	-0.246
CsAOXa*	294	7.06 / 5.56	34.2 / 28.7	99.69	49	-0.145
CsAOXb	284	6.60 / 6.07	32.2 / 28.8	93.36	31	-0.310
CsAOXc	335	8.60 / 6.49	37.9 / 32.7	98.98	48	-0.381
CsAOXd	319	8.29 / 6.49	36.4 / 33.7	81.90	24	-0.191

All proteins showed phosphorylation sites: CcAOXb has 40 phosphorylation sites (9Thre/26Ser/5Tyr); CcAOXa has 36 phosphorylation sites (11Thre/21Ser/4Tyr); CsAOXa, CsAOXa* and CsAOXb have 34 phosphorylation sites (10Thre/21Ser/3Tyr, 9Thre/19Ser/3Tyr and 7Thre/24Ser/3Tyr, respectively); CcAOXd has 31 phosphorylation sites (11Thre/18Ser/2Tyr); and CcAOXc, CsAOXc and CsAOXd have 30 phosphorylation sites (14Thre/14Ser/2Tyr, 14Threo/14Ser/2Tyr and 11Thre/17Ser/2Tyr, respectively; Table 3; S3 Fig). Only CcAOXb, CcAOXc, CsAOXb and CsAOXc proteins showed N-glycosylation sites (1, 2, 1 and 2 sites, respectively; Table 3; S4 Fig). The pfam01786 functional domain was found in all citrus AOX proteins (S4 Fig). As previously suggested [46], AOX regulation might also occur via phosphorylation of the N-terminal extension through charge-induced conformational changes and/or an interaction with other mitochondrial proteins. The protein sequence identity varied from 28% to 78% between CcAOX proteins and from 26% to 97% between CsAOX proteins (S5 Fig). The greatest degree of identity was observed between CcAOXd and CsAOXd (99%), CcAOXc and CsAOXc (99%), CcAOXb and CsAOXb (94%) and CcAOXa and CsAOXa (98%) (S5 Fig), and for this reason these gene pairs could be considered orthologues. The percentage of identity between the two proteins resulting from the alternative transcripts of the gene *CsAOXa* was 97% (S5 Fig).

**Table 3:** Post-translational modifications of citrus AOX proteins. * Protein resulting from the alternative transcript of the *CsAOXa* gene.

Protein	Phosphorylation sites	N-glycosylation sites
CcAOXa	T_4_, T_8_, T_27_, T_30_, T_81_, T_127_, T_198_, T_223_, T_261_, T_292_, T_343_, S_6_, S_10_, S_13_, S_21_, S_37_, S_38_, S_41_, S_43_, S_66_, S_85_, S_97_, S_162_, S_205_, S_213_, S_218_, S_267_, S_304_, S_307_, S_309_, S_319_, S_342_, Y_119_, Y_154_, Y_202_, Y_276_	-
CcAOXb	T_3_, T_7_, T_29_, T_88_, T_119_, T_144_, T_188_, T_241_, T_272_, S_2_, S_13_, S_25_, S_27_, S_30_, S_36_, S_47_, S_48_, S_50_, S_51_, S_52_, S_53_, S_54_, S_55_, S_56_, S_57_, S_58_, S_59_, S_60_, S_61_, S_161_, S_168_, S_221_, S_239_, S_251_, S_271_, Y_4_, Y_117_, Y_240_, Y_264_, Y_293_	N_22_
CcAOXc	T_12_, T_14_, T_20_, T_31_, T_37_, T_52_, T_131_, T_132_, T_141_, T_166_, T_210_, T_263_, T_284_, T_294_, S_11_, S_19_, S_36_, S_47_, S_51_, S_88_, S_134_, S_144_, S_183_, S_190_, S_243_, S_261_, S_293_, S_329_, Y_116_, Y_286_	N_49_, N_292_
CcAOXd	T_26_, T_104_, T_120_, T_121_, T_130_, T_138_, T_155_, T_199_, T_252_, T_283_, T_289_, S_8_, S_20_, S_52_, S_53_, S_55_, S_56_, S_57_, S_58_, S_60_, S_77_, S_92_, S_110_, S_172_, S_179_, S_232_, S_250_, S_260_, S_262_, Y_54_, Y_128_	-
CsAOXa	T_4_,T_8_, T_27_, T_30_, T_81_, T_127_, T_198_, T_223_, T_261_, T_292_, T_343_, S_6_, S_10_, S_13_, S_21_, S_37_, S_38_, S_41_, S_43_, S_67_, S_85_, S_97_, S_162_, S_205_, S_213_, S_218_, S_267_, S_304_, S_307_, S_309_, S_319_, S_342_, Y_119_, Y_154_, Y_202_	-
CsAOXa*	T_4_,T_8_, T_27_, T_30_, T_81_, T_127_, T_198_, T_223_, T_261_, S_6_, S_10_, S_13_, S_21_, S_37_, S_38_, S_41_, S_43_, S_67_, S_85_, S_97_, S_162_, S_205_, S_213_, S_218_, S_267_, S_288_, S_289_, S_290,_ Y_119_, Y_154_, Y_202_	-
CsAOXb	T_15_, T_74_, T_105_, T_130_, T_174_, T_227_, T_258_, S_11_, S_13_, S_16_, S_22_, S_33_, S_34_, S_36_, S_37_, S_38_, S_39_, S_40_, S_41_, S_42_, S_43_, S_44_, S_45_, S_46_, S_47_, S_147_, S_154_, S_207_, S_225_, S_237_, S_257_, Y_103_, Y_226_, Y_250_	N_8_
CsAOXc	T_11_, T_13_, T_19_, T_30_, T_36_, T_51_, T_130_, T_131_, T_140_, T_165_, T_209_, T_262_, T_283_, T_292_, S_10_, S_18_, S_35_, S_46_, S_50_, S_87_, S_133_, S_143_, S_182_, S_189_, S_242_, S_260_, S_291_, S_328_, Y_285_, Y_314_	N_48_, N_291_
CsAOXd	T_20_, T_98_, T_114_, T_115_, T_124_, T_132_, T_149_, T_193_, T_246_, T_277_, T_283_, S_14_, S_46_, S_47_, S_49_, S_50_, S_51_, S_52_, S_54_, S_71_, S_86_, S_104_, S_166_, S_173_, S_226_, S_244_, S_254_, S_256_, Y_48_, Y_122_	-

**Fig 3:**
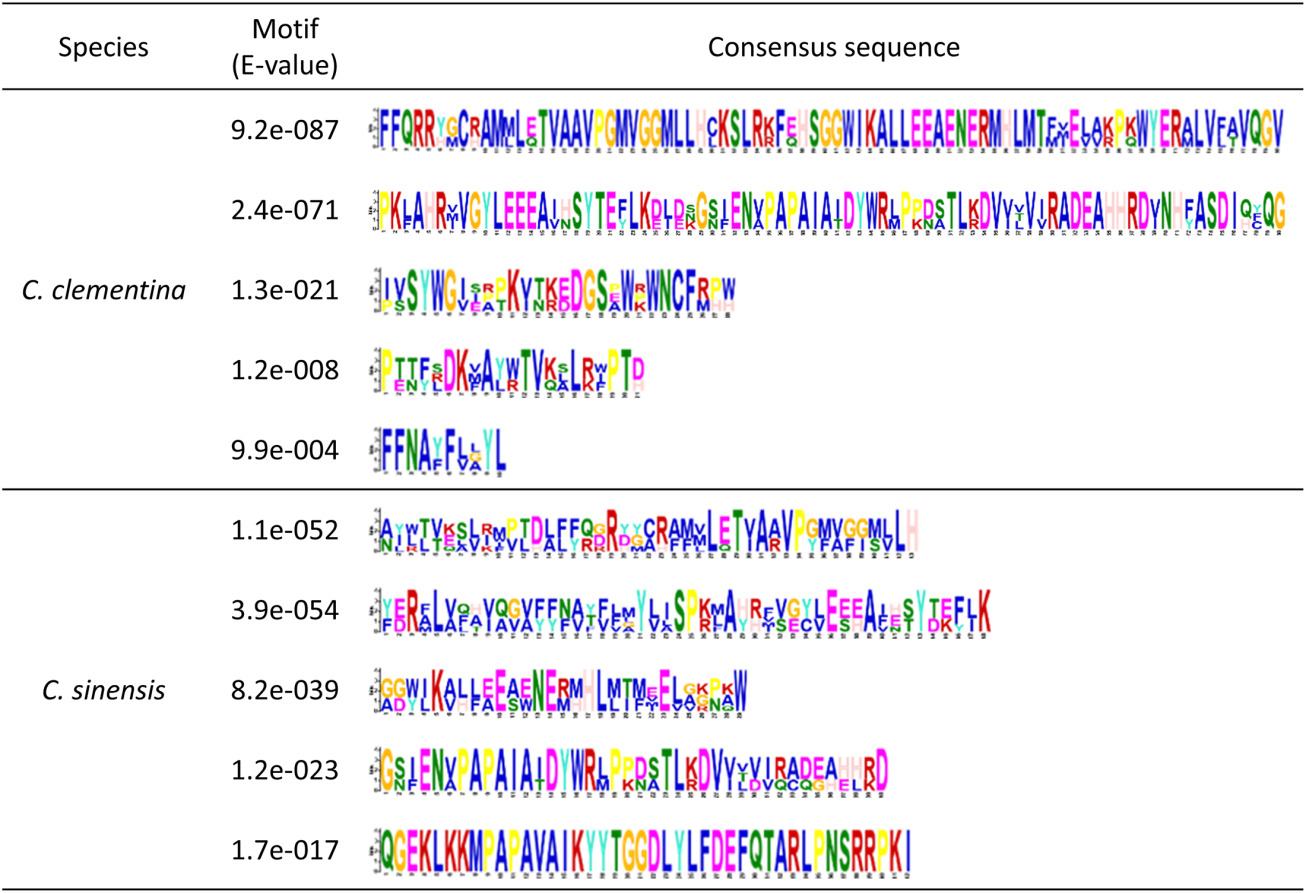
Conserved motifs in citrus AOX proteins obtained by the MEME program.

The motif analysis of the predicted citrus AOX proteins by the MEME program showed that the mandarin and orange AOX proteins contained the typical LET, NERMHL, LEEEA and RADE-H conserved motifs (Fig 3; S4 Fig). These motifs were found in AOX proteins from other plant species [47]. The hydropathicity analysis revealed a profile with two hydrophobic regions for all the citrus AOX proteins (data not shown).

### Phylogeny analysis

**Fig 4:**
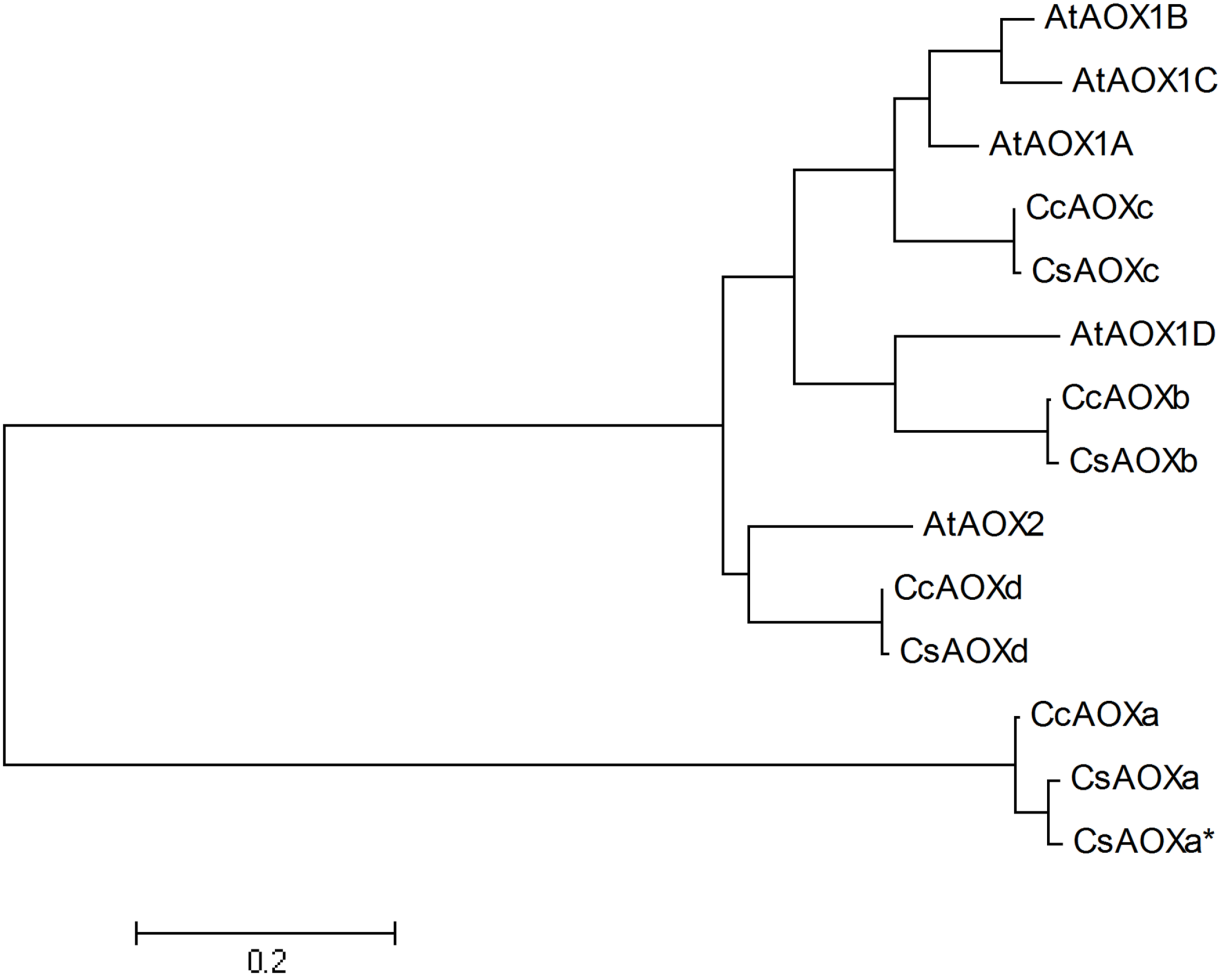
Phylogenetic tree obtained with the AOX proteins of *A*. *thaliana*, *C*. *sinensis* and *C*. *clementina*.

Phylogenetic analysis of the AOX citrus and *A*. *thaliana* sequences showed that the CcAOXb and CsAOXb were closed to the AtAOX1D sequence while CcAOXd and CsAOXd were closed to AtAOX2 (Fig 4). The CcAOXc and CsAOXc sequences were grouped with three *A*. *thaliana* sequences AtAOX1A, AtAOX1C e AtAOX1B (Fig 4). The CcAOXa, CsAOXa, CsAOXa* constituted a separated group in the phylogenetic tree, without proximity with the *A*. *thaliana* sequences. The comparative analysis of the citrus and *A*. *thaliana* sequences did not allowed a clear classification of the citrus AOX sequences in relation to *A*. *thaliana* ones, mainly in the case of AOXa, AOXa* and AOXc.

### Molecular modeling of CcAOXd protein and docking with the ubiquinone

**Fig 5:**
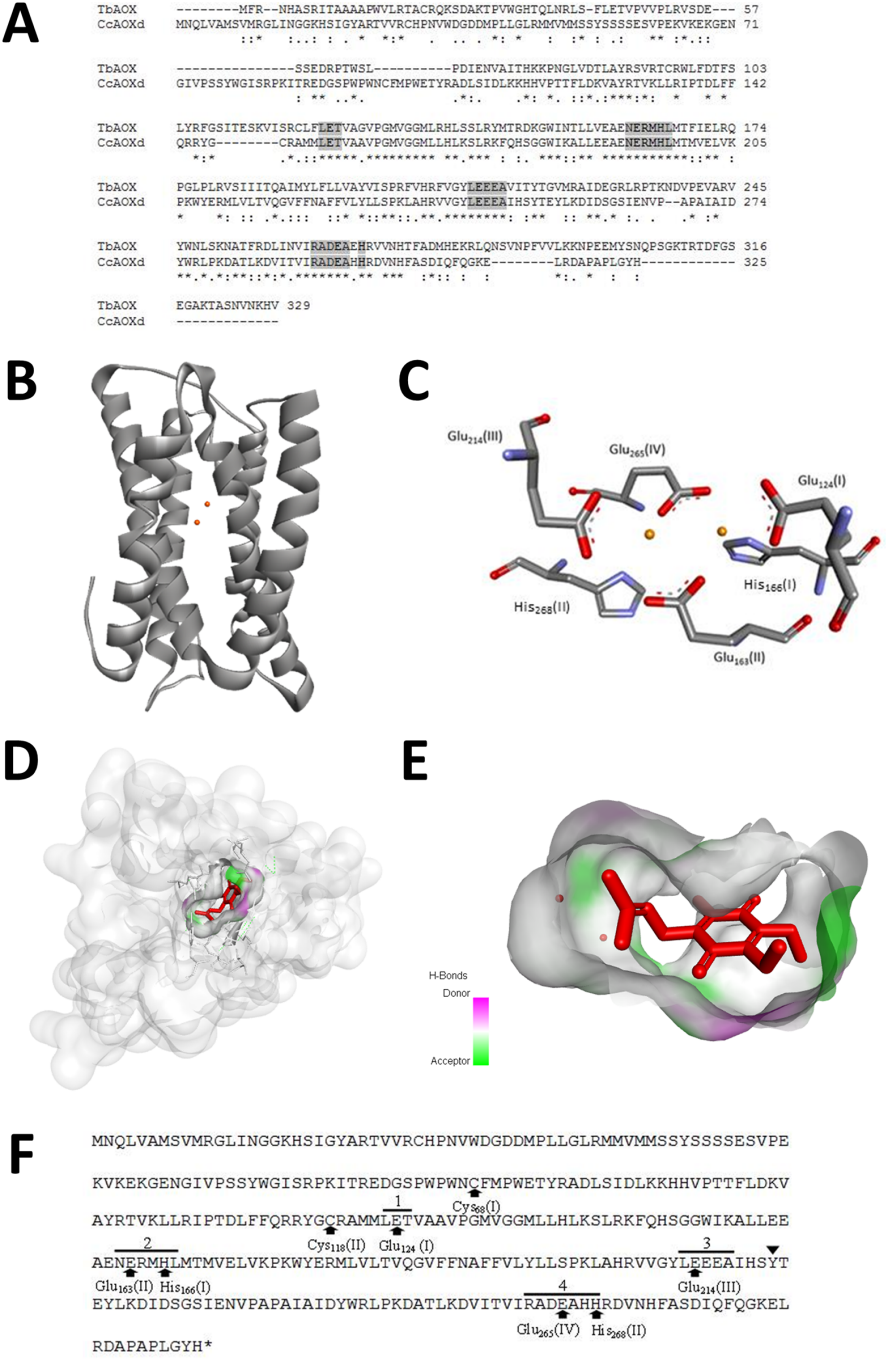
Tridimensional structure of CcAOXd obtained by homology modeling with the *T*. *brucei* AOX (Pdb code 3VV9) as a template. **A.** Alignment of TbAOX and CcAOXd proteins. Gaps introduced to get the best alignment are indicated by (-). Highly conserved domains related to protein structure and activity are indicated in grey. Identical amino acids are indicated by an asterisk (*), conservative substitutions by a colon (:) and semiconserved substitutions by a period (.). **B.** Representation of the 3-D structure of CcAOXd (in grey) containing 6 helices. Iron atoms are represented by orange spheres. **C.** Structural details of the CcAOXd catalytic center, showing the CcAOX diiron center. The diiron center contains 4 Glu and 2 His residues. Iron atoms are represented by orange spheres. **D.** Molecular surface position of the hydrophobic cavity during docking with UQ. UQ is shown in red. **E.** Structural details of UQ occupying the hydrophobic cavity. Iron atoms and UQ are represented by orange spheres and in red, respectively. Donors and acceptors H-bonds are indicated by a purple and green color gradient **F.** Predicted CcAOXd peptide sequence showing conserved domains and structurally important amino acids. The black triangle indicated the position of the redox-active Tyr (Y), and the 4 iron-binding sites are numbered from 1 to 4. The black arrows highlight the Glu (E) and His (H) residues, which are important for the coordination of the diiron center.

The best alignment of the citrus AOX proteins with the Pdb was obtained between the CsAOXd and CcAOXd protein orthologues and the AOX protein from *Trypanosoma brucei* (TbAOX, PDB ID: 3VV9, MMDB ID: 108244). The protein CcAOXd was chosen for the molecular modeling and the subsequent docking. The alignment of the amino acid sequences of CcAOXd and TbAOX presented 68% coverage, 45% identity (E-value 7e-55) and an RMSD of 2.85 Å (Fig 5A); these values (identity >25%) indicate that the TbAOX protein is a good model to be used as a template [48]. The validation analysis (Ramachandran plot) of the CcAOXd model showed that 92.9% of residues was in most favored regions and 5.7% was in additional allowed regions, indicating that 98.6% of the amino acid residue was located in favored regions (S6 Fig). In addition, ANOLEA showed good energy values as well (S6 Fig). The 3-D model of CcAOXd showed a total of six helices, two of them anchored in the inner membrane of the mitochondria, and the other fourth helices–rich in histidine and glutamate–were in contact with the mitochondrial matrix (Fig 5B). The first transmembrane helix has 21 amino acid residues in the positions 150–170, and the second has 20 residues in the positions 112–131 (Fig 5A and 5B). The length of the two transmembrane helices is compatible with the length required to cross the mitochondrial membrane. The largest portion of the CcAOXd protein remained in contact with the mitochondrial matrix, with only few residues anchored in the mitochondria membrane, which explains the negative values of hydropathicity, typical of cytoplasmic proteins (Table 2). CcAOXd presents 4 highly conserved domains (LET, NERMHL, LEEEA and RADE-H; Fig 5A, S4 Fig) that contains histidine and glutamate residues responsible for the interaction with the iron atoms; all these elements constitute the di-ferric center of the AOX enzyme (Fig 5C) [46, 49]. This association with iron atoms classifies the AOX proteins as belonging to the R2 subunit of ribonucleases [46, 49]. Two cysteine residues [C_68_ (I) and C_118_ (II)] that are conserved in the AOX proteins of different plant species and assumed to be involved in the redox regulation of AOX activity were identified in the CcAOXd structure (C_68_ (I) and C_118_ (II); Fig 5D). C_68_ (I) and C_118_ (II) also play a role in the post-translational regulation of most angiosperm AOX proteins [50]. The CcAOXd structure contains a redox-active Y_221_ that is highly conserved across other AOX proteins [47, 51] and that could play a key role in the AOX catalytic site (Fig 5F). The active site, which is located in a hydrophobic environment deep inside the CcAOXd molecule, is composed of the diiron center as well as 4 glutamate (E_124_, E_163_, E_214_ and E_265_) and 2 histidine (H_166_ and H_268_) residues, all of which are highly conserved among AOX proteins (Fig 5F). Molecular docking results presented an affinity energy of -7.0 Kcal/Mol and indicated that UQ bound to CcAOXd in a recessed pocket formed between the helices and submerged into the membrane (Fig 5D and 5E); the pocket is formed by Arg_105_, Asp_109_, Arg_119_, Leu_123_, Glu_124_, Ala_127_, Glu_163_, Leu_213_, Glu_214_, Glu_216_, Ala_217_ and Glu_265_ amino acid residues. The 2-D map of the interaction between CcAOXd and UQ showed the van der Waals, carbon hydrogen bonds and alkyl interactions, among others, which related the CcAOXd proteins to UQ (S7 Fig). As in TbAOX, this second cavity connects the diiron active site with the outer mitochondrial membrane and interacts with the inhibitor-binding cavity at the active site [52].

### *In silico CsAOX* gene expression

**Fig 6:**
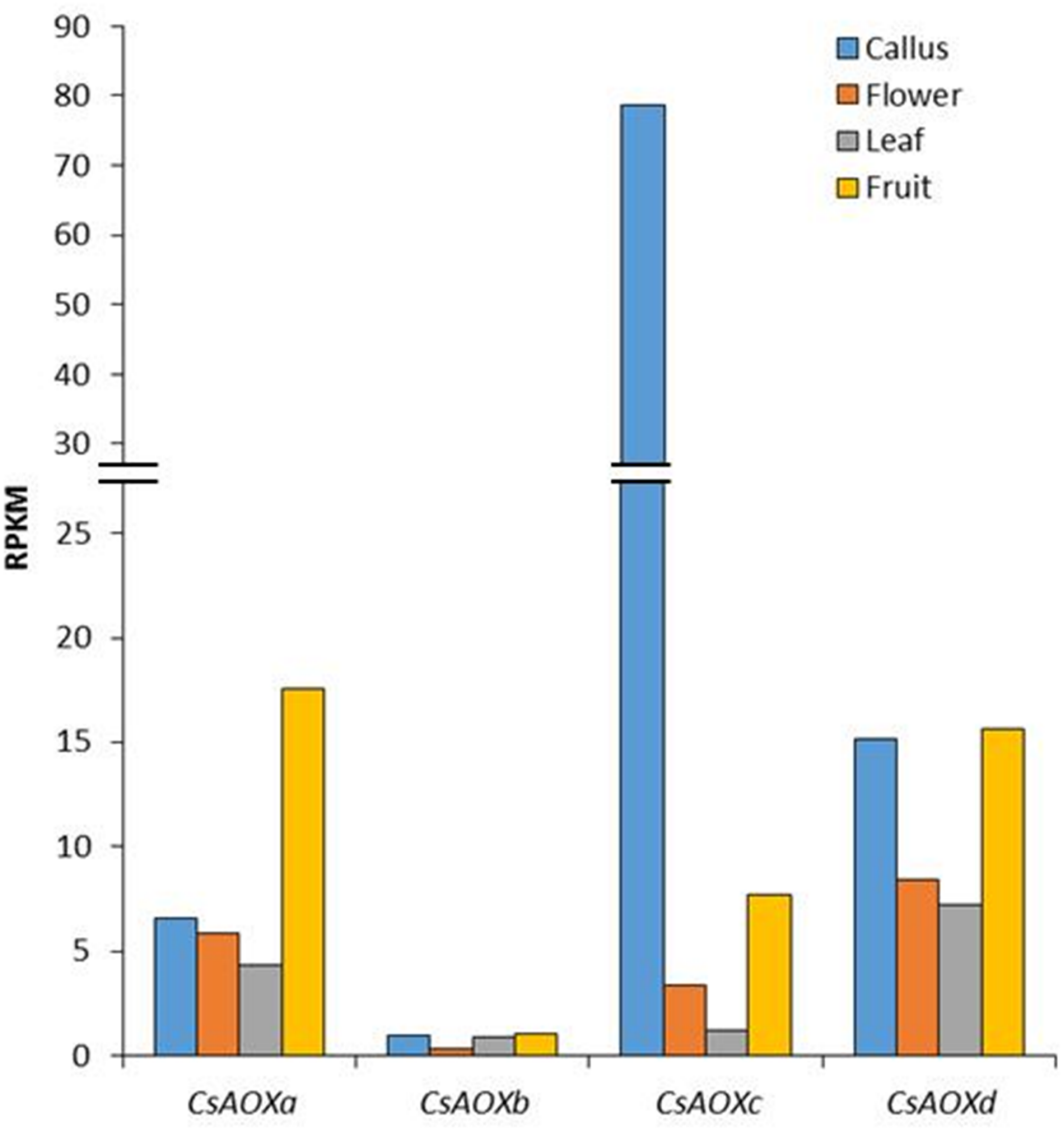
Expression of *CsAOX* genes in different *C*. *sinensis* tissues.

The expression of the *CsAOX* genes was previously obtained and was available in the CAP database [31]. Four tissues were analyzed: callus, flower, leaf and fruit (Fig 6). The *CsAOXa*, *CsAOXc* and *CsAOXd* showed high expression levels (>3 Reads Per Kilobase Million/RPKM excepted for *CsAOXc* in leaf) while the *CsAOXb* was lowly expressed (<1 RPKM) (Fig 6). The *CsAOXa* gene was highly expressed in the fruit (17.5 RPKM) but also showed significant expression levels in callus, flower and leaf (6.6, 5.9 and 4.3 RPKM, respectively; Fig 6). The *CsAOXc* gene showed the highest expression level in callus (78.5 RPKM) and significant expression levels in fruit and flower (7.68 and 3.37, respectively; Fig 6). The *CsAOXd* gene presented similar expression in callus and fruit (about 15 RPKM) and also close values of expression in flower and leaf (8.4 and 7.2, respectively; Fig 6). These results showed that the *CsAOX* family members were spatially differentially expressed among citrus organs; some similar results were previously described in *A*. *thaliana* [53, 54]. The very high expression of the *CsAOXc* in callus could be correlated with high expression level of *AtAOX1A* and *AtAOX1C* –both phylogenetically closed to *CsAOXc* (Fig 4)–in chilling-stressed callus [53]. The relatively high expression of *CsAOXa* and *CSAOXd* in fruits (>15 RPKM; Fig 6) may be related to the expression of *AOX* genes from other species producing fruits such as tomato, papaya or mango [55–58]. Some *AOX* genes were related to fruit maturation, ripening and post-harvest ripening in association with ethylene peak emission (climacteric fruits) [56, 57], while other *AOX* genes were associated to gametophyte development [58]. Some *AOX* genes related to climacteric fruit ripening presented elements responsive to ethylene in their promoter sequences [57]. Here, the *CsAOXa* and *CSAOXd* genes did not present any elements responsive to ethylene in their promoter sequences; this could be related to the fact that citrus are non-climacteric fruits, or may suggest an involvement of these *CsAOX* genes in fruit formation more than in fruit ripening (Figs 2 and 6).

### Conclusion

To the best of our knowledge, this is the first characterization of the *AOX* gene family in *C*. *clementina* and *C*. *sinensis*. Four *AOX* genes were identified in each species; the *C*. *clementina* genes were orthologues of the *C*. *sinensis* genes. Phylogenetic analysis of the AOX citrus and *A*. *thaliana* sequences showed that the CcAOXb and CsAOXb were closed to the AtAOX1D sequence while CcAOXd and CsAOXd were closed to AtAOX2. According to the *cis*-element present in the citrus *AOX* promoters, the gene expression may be regulated by several external or internal factors. Expression of *CsAOX* genes revealed that *CsAOXc* was highly expressed in callus while *CsAOXa* and *CsAOXd* were highly expressed in fruits. Other regulation levels were also predicted, such as alternative splicing and post-translational modifications. The corresponding proteins were predicted to be directed to the mitochondria, and the analysis of the 3-D structure of one the *C*. *clementina* AOX isoforms showed the presence of two hydrophobic helices that may be involved in the anchoring of the protein in the inner mitochondrial membrane. The active site of the protein is located in a hydrophobic environment deep inside the AOX structure and contains a diiron center. The molecular docking of CcAOXd with UQ showed that the binding site is a recessed pocket formed by the helices and submerged into the membrane. These data are important for future functional studies of citrus AOX genes and/or proteins, as well as for biotechnological approaches leading to AOX inhibition using UQ homologs.

## Supporting information

S1 Fig Nucleotide sequences of *AOXs* from *C*. *clementina* and *C*. *sinensis* from the Citrus Genome Database. (DOCX)

S2 Fig Promoter sequence of the citrus *AOX* genes (1500 bp upstream, except for CsAOXd). (DOCX)

S3 Fig List of the *cis*-elements found in the promoter regions of the citrus *AOX* genes. (DOCX)

S4 Fig Amino acid sequences of AOX from *C*. *clementina* and *C*. *sinensis*. (DOCX)

S5 Fig Amino acid sequence identity of CcAOXs and CsAOXs. (DOCX)

S6 Fig Modeling validation of the CcAOX structure using the Ramachandran plot and the ANOLEA analysis. (DOCX)

S7 Fig 2-D map of the interaction between CcAOXd and UQ. (DOCX)

## Abbreviations

AOX: alternative oxidase
MW: molecular weight
ORF: open reading frame
pI: theoretical isoelectric point
RMSD: root-mean-square deviation
RPKM: Reads Per Kilobase Million
UQ: ubiquinone
