# Supplementary material for: Alternative oxidase (AOX) constitutes a small family of proteins in Citrus clementina and Citrus sinensis L. Osb
Source: PLoS One. 2017 May 1;12(5):e0176878. doi: 10.1371/journal.pone.0176878 (PMC5411082; doi:10.1371/journal.pone.0176878)
Supplement: S1 Fig — (DOCX) [file pone.0176878.s001.docx]

**S1 Figure.** **Nucleotide sequences of *AOXs* from *C. clementina* and *C. sinensis* from the Citrus Genome Database.** 5’ and 3’ UTR are indicated in green and exons in red. Intron are indicated without any colour.

CcAOXa

>clementine0.9_012574m scaffold_19:638155..642108 +

TGATGACAACTCTCTCTCAAACAGTATCGGTCTTTTCCAATAAAGCAATA

AATCTTAAAAGCGTAGCTTCTTTCAACACCATCAAGACTTTGCGTTTCAA

CCCTCCCTCTTCACCTCGATCGCTATCCAGGTTAGTTTTCAAACGATTAT

CTCTAATAATTTTTTTTGAACTTAACTTATCCGTGAATTTGTTTTCTGTA

ACTTTGTTTCAGGAAGTTTTGTCGAGTTCAAGCCACAATTTTGCAAGATG

AAGAGGAGAAAGTGGTGGTAGAGGAGTCATCTCCACTCAAGAATTTTCCA

AATGATGATGAGCCACCCGAGACTGGGTCAGCTAGTGCATTGGAAAAATG

GGTTATCAAGTTTGAACAATCTGTTAATATCTTACTTACGGTAAATCAAG

TGAAGTCTTTTGTAGCTTAAGTGATGCGGTGCTTGGTGTTGCAAGATTAT

TAACTCTATTAATCATTTCAGGAATCTGTGATAATGGTACTTGATGCTTT

GTATCGTGACCGGGATTATGCAAGGTTCTTTGTACTGGAGACTATTGCTA

GAGTTCCTTATTTTGGTGAGTGTTGTTCTTTCTTTACATTTTTACCCTTA

TTTGGTTTCGACGGCATAATTCATCTGGCATAATCCATCATTTAGCATTT

GTTGTTTGAAGGTGATTGAGGGCCCGTTTGGTGTTTGGTATCGAGGTGCT

GTTTTTTTTTTTTTAGTTTGGGGGGGGGGGGGGGGGGGAAAGGGAACCTT

TATTTTGAAATAATAGTTGATAGTGTGTGACTGTGTGTGTGTGTATGACT

TTCAAATAATAATATTAACAACAATAAGAAAAGATTAATTTTTCATCTTA

CAAGTGTAGGATTAAATCAACCTAACTTTCAAATTACAGAGGTAGGTATT

TACCCAAATACGTTAGTATTGTTGGCTTTTAACCAATAGTTGCCCAACGA

TAATACCAAACTGGACCTTAATTTGATATAGCATCTTGGTAAGTGGGGGG

CCCTTTAAATGTTATTTCAGTTTTACTCGCTTATTAAAGGATTTTTCTTC

TGTGGTCATGCCGCTATTAGTTGTGACTTATGGTTTCTACTGCAGCATTT

ATTTCTGTTCTGCACATGTATGAGAGTTTTGGTTGGTGGAGAAGAGCAGA

TTACCTGAAAGTTCATTTTGCTGAGAGCTGGAATGAGATGCATCACTTGC

TCATAATGGAAGTAAGGCTCTGTTATTAGATGTTTGTAATACTTTCATGT

TGTACTCTTATAAAACAGGTTAGCTGAGAGGCTTAACCTGTTACTTATGG

CAGGAAGCCTATTACTGTATCTTGTCATGTTAATTTCTTTACTATCTCCT

GTTCTATGATGCATATCTTGGTTGAAGTTTTCTTAATCCAACTAGCAGGA

AATCTAACATCTTTGAACTGAAGGCAACGGCTTAAGACTATAGTGGATTG

AGGAAAATGGATAGCACCTAATGAGACACTCATTGGAGGAATTTGAAATT

ACGTTGCTCCTCCCTATCGTACCCCAAAAAAAAAAAAAATTACGTTTCTC

TGCTTAAGTTGCAAAAGATATCACTAATAGTTTTCTGTCTCTTAATCTGA

TCCAACCACCTTGCCTATTCAGGTCCAAAATAATATGAAACATTTAGACA

TTACTTGCACATTTGTTTTGTTTCTTAGTCATTTAGTCATTTGGTTGCAG

GAATTGGGAGGAAATGCTTGGTGGTTCGATCGCTTTCTTGCGCAGCATAT

AGCCGTTGCTTATTATTTTGTGACAGTCTTCATGTATGTAATAAGCCCAA

GAATGGCATGTAAGTATTTGCATTGGGATTTTTAATATTAAAGTAGAACC

TCGTTAGATTAAAATTCAATAAATTTATAACGTTGATTAAATAATAATTT

TTGTTCGTCTTGATTATGGACCAATGTAGTAAATCAATAAACAATAAATT

TATTACATAATAAATTTTTTAAATTTCTATAGGGCCCAATTTATACATAA

ATTAATAATTAACTAATTTGAAACTAAACACGTATGATAGGTTTATGATT

TTTTTTTTTTGGTACTAGCATAATGCTTTACGCATTTCATCTGTCATTGT

TGTTTTCTCTACACTTTTCAAATAAAAAGTCATTTTTGTATTTTATGTGC

TATGTTAAGAGAAATAAGAGAGAGACTAACTTAATATATTGCCTCAAAAA

AGTCTTCTGTGATAAGATACTATATAACTGTGATCCAGTCATGCTATCCA

ACCAAAGGATTATCAAGTGACTTATTTGCTCAATCTGGATCAAATCGAAA

TTGTATCATGCAACTTGACAGAATCACTGCATTCTCTAATTATACTATCG

TTGTACTTTATAAAAATTCTAATTTTGAAAGTAATATTATCTTTTAATAT

TACTTAAATTAATAGATTTTTCATGGTCCCGCAAGGCTATTAATTTATCG

AGGTTTTACCATATATTCCTAGATTTTCTCATGCTTACTAGAATCTTGTT

ATTTTAGTCATCCCTGAATGTTTATAAATCTTTTTCCTTCATTTGATGCT

TTTCCCATGCAATGCTCCAAGGTATTTTTGAATTTCAAATAGAACAAGTT

CGTTTAAGAACAAGCTAGTATGTTTATGTTTCATCCAACTGCAGATCAAT

TTTAATGTATTCATTTTATTGCTTGTGCAGATCACTTTTCGGAATGTGTG

GAGAGCCATGCTTTTGAAACTTATGACAAATTTATCAAGGCCCAAGGAGG

TAAAATTTTTTTAATGTTTTGGAAAGCTTAGTTTGATTGTCTTGCGCATA

TACTTCTATTTGACATTGAATTGAAGCAAAATAGTTAAATGATTCCTATC

TTGCTTACAGTTCTCATGGTACATTTTTTGGCCCTGATAATGCCACCAAG

CAAGTTCAATTCTCATATAACACTGGCTATGTAAATCTGATGACCTCTAC

TCTGAGGATGCTAGTTAATCGGCCGTTAATTTTTTCAAACGCGAACGGAT

AATTGGTGTCTTTTAAGTATGGATTAGTGTGATCAAATGAACTTCATATT

CTGCATGTGTAGTACAGTCCATTTTCACTAGGTTTCACCTGCTCTGTCCC

CTCTTTTTACAGAGAAGTTGAAAAAGATGCCTGCACCGGCGGTTGCCATT

AAGTACTATACTGGAGCCGACTTGTACTTGTTTGGTAAGTTTGTCTTCTT

GCAACCTTTATAACTGTCATGTAAAACAAGATCTAGCTTTTAACTGAGTC

ATCTTCATTTCTACCCAGATGAATTTCAAACTGCAAGACTCCCAAATTCT

CGAAGACCAAAAATAGGTAAAGAACGTGTACTGTTTCTAGCACTGCCTAT

AAGAAGCTACTCAAGTTCTATACAACAATACTAAATTGCAACTTGCCGGA

CATACATTATGAATACATATTGACGGAACTTGAAATATCTTTCTGATTTG

TTTGTTTTCAGAGAATTTGTATGATGTGTTTTTGAACATTAGAGATGATG

AAGCTGAACATTGTAAGACGATGAAGGCTTGTCAGACACATGGAAACCTT

CGGTCACCTCATTCTTATTCAGAGGATGATTTTGGTTGCGAAGATGAGTC

TGGCTGCATAGTTGCTCAAGCAGATTGTGAAGGAATTGTAGATTGTATAA

AGAAAGCTGTAGCTGTTAGTACTCCCCGAGTAAAGCAAAACTAAGCTTTT

CTCTTTTTCTTTATTTTAGAAGGAAGAATGTGATGAAAATTGGCTTAGAA

TTTACCAACAGTTACAGGGCTGTGTTGTATACACAAAAAGAAACCATATA

GATCCATACAAAGTTAATCTACACAGGTTTTCTG

CcAOXb

>clementine0.9_034013m scaffold_1:3771541..3772917 -

ATGAGTACTTATCGCGCCACGGCACGTATATTGAGGTCGTTGATGTGGCA

AGCAGCTGGGGCTAATTATTCCTCCTCTTCATTAACCTCGGGGAGGCATC

TGATGAGTCGTTATCCCGCGGGGATAGTGCGGTATTGGAGTAGTGCTTCA

TCATCATCATCATCATCATCATCATCATCATCAGCTCCTCCTGTAGACTT

GCCGAAGGACAAAGAGGAGATCAATCAGCAAGTGTCAAGTGCAAGCCCTT

CCGATCATAAGCAAATGTTAAGAACATTGTTAGCTACTGGGGCATCATTC

CAACTAAGGTTACCAAAGAGGATGGCTCTGCCTGGAGGTGGAATTGCTTC

AGGGTAAGTGTACATACACATTTACGCAAGCTCACGAGTAATAATTAATA

ATATTACATGTTCTAATCAAGTGGGTAAATTCACTATCAGTTACTAGCTA

GGGTCTGGTATGAATTCTCAGTTGATCCTCGTGTCATTTGAGCCATAGCG

ATGTGATCTACCATTATAATAATTCAAATGATATTGCATTCATCGAATAA

TAACTTGAATGATCGGTGTTATTATTGCAGCCCTGGGAGACCTAAAAAGC

CGACTTGTCAATTGATGTCACCAAGCACCACAAGCCAGAAAATTACAGGG

ACAAATTTGCATACTGGACAGTCCAAGCTCTCAAATTCCCAACCCATTTA

TTCTTCCAGAGACGCCACATGTGTCACGCCATGTTGCTCCAGACGGTGGC

CGCCGTCCCCGGAATGGTGGGCGGCATGCTCTTGCACTGCAAGTCACTGA

GGAAATTCGAACACAGCGGCGGCTGGATCAAGGCTCTGTTGGAAGAGGCA

GAAAATGAGCGCATGCATTTGATGACATTCATAGAATTGGCCAGGCCTCA

GTGGTACGAGCGAGCCCTGGTGTTTGCCGTACAAGGAGTGTTTTTTAATG

CTTATTTCTTGGCATACTTGGCATCTCCAAAGCTCGCTCACCGCATTGTG

GGTTACTTGGAAGAGGAGGCTGTGAATTCTTACACTGAGTTTCTTAAAGA

CTTGGAGAACGGCAGCTTTGAAAATGCTCCGGCTCCGGCTATTGCCATTG

ATTATTGGCGTATGCCTCCTGATTCAACACTTCGTGATGTTGTGGTGGTC

ATACGAGCCGATGAGGCGCATCATCGGGACATTAACCACTATGCTTCGGT

AAGAATCGAACTAAAACACACACACACACACACACACAAATTAACGGTTC

TATTTGTTTGTTTAAATATTTTTAATTGATAATTTTGGTTGTTTGTGTTG

CAGGACATTCAATGTCAAGGGCATGAGCTCAAGGACGCACCTGCTCCGGT

TGGATATCATTG

CcAOXc

>clementine0.9_015158m scaffold_1:3767033..3769080 -

AAGAAACAACAAAAGCAGCCAAAACTCTTACCAAATTAACAAATCAATAG

CAGTCATTTCTTTTCGTCGATCCTTTAATTCTTGAACATGATGTATCGTG

GTGGGGTCCGCTTGTTTTCTACCCTAACCGCACGCACAGCTTCAACCGAG

GCGGCCGCCAGGATCTTGGCCGGCCAAACTAACAGGCATTTATCGACCTT

CTTGGTTAGGGCTCCGATTCTAGGCTCAAGGAACCGGAGCACGTTGGCTT

TAGGTGGTGAGAAAGAACAACAGCGGGAGGAGAATGTTCAGACGACCGGT

GCCGCGGCGGCCGGTGGCAGTGGGAACAAGGATGAGAAGAGGATTGTGAG

TTACTGGGGCGTGGAGGCTCCTAAAGTTAACAAAGATGATGGCAGTGAAT

GGAAATGGAACTGCTTTAGGGTATATGCTCATGAATTGTTATTATTAATT

TCTTTTCTTGGATCTTTATTTTACGTGTGCTTTAAGCAAATCATTGATGT

GTCTGTGGTGTTTGTGTTTATATTATTATAGCCATGGGAGGCTTACGAAG

CGGATTTATCAATTGATTTGAAGAAGCACCACGCACCGACGACCTTTTCG

GATAAAATGGCCTTGTGGACCGTCAAATCACTCAGGTGGCCCACTGATTT

GTTCTTTCAGGTATATATTTGTTGTTATGTTTTCGATTGATCAGGGGAGT

GGCCGAATAGGGATTTTGGTTCGGGGCAGGGAATTAAATATAGAAACATG

AAACATGAAGGGGTTATTCATAATATCTGTAATTGTAGGGAAAAATAAAT

TGATAAACAATTAAACACTAACCAATTTCTTTTTCTTTTTTTTTTTAGAA

ACGATTAATAGAAATATAATTTTGTATTAATAAGGATTTTGGCTTGATGC

TTTAAATATAAAAATATTATACTTGATTGAGTCTACGACATAATATCTGT

AAAATTTAGGGGCCAAATATAAACTTTTCATAGAAATTGACTAAATTTCA

GTTGAATCCCTCAATAATTTCTGAATTTTGGTGAAATTGGTAAAGATTAA

TAAAAACTCTTTTTTATAATTAATTTGAACAGAGAAGATACGGGTGCCGG

GCTATGATGCTCGAAACAGTGGCGGCAGTCCCCGGGATGGTGGGAGGGAT

GCTGTTGCATTGCAAGTCATTGAGGCGTTTCGAGCACAGCGGTGGTTGGA

TTAAGGCTTTGTTGGAAGAAGCAGAGAATGAACGAATGCATTTGATGACA

TTCATGGAGGTAGCAAAGCCTAAATGGTACGAGCGTGCTCTTGTGTTTGC

AGTGCAAGGTGTGTTCTTCAATGCTTACTTCTTGGGCTATCTCATTTCGC

CTAAGTTCGCTCATCGGATGGTTGGGTACCTTGAGGAAGAAGCAATCCAC

TCGTACACCGAGTTCCTCAAGGAGTTGGATAAAGGTAACATTGAGAACGT

TCCTGCTCCGGCCATTGCCACTGATTACTGGAGGTTGCCTCCGAACTCGA

CTCTCAAGGATGTTGTGCTGGTTGTAAGAGCAGATGAGGCTCACCACCGT

GATGTTAATCACTTTGCATCGGTATGATCAGGATTCTCTTTTGAGCTAAA

CTATTTGCAGGATACATTTGTTCGGCAGTAACTTTTAATACTTGCCTTTT

TGAAATGCAGGACATACATTACCAAGGGCGCCAATTGAGGGAATCTCCAG

CTCCACTGGGGTATCACTGATCCAATGATGAAAAGAATCAGAATTCAAAT

ACAACATGAATTTTACTACGGTGGTAGTATTGGTAACAATAACAATAAAA

CCCTTTTGAGTATACGAATAGTATTTGTTCGCTTTGTAAATGTTTGTGGA

GTAACTGAAGTTTGTGGTCCTTTGATGTTTGTTAAGAGTCGAGTCTGTTT

AAACACGAGTTTGAAGCAGCATATGAATTGTTATTTTTCTATTAAAAGAG

CCTTATTATTCCTATGCCATCGAAATTGGTAGATGTATGCGACCACCTGT

AAAGATTTATTCTGCACCTAATTGTTAATGAGTTTATTATATCCCATG

CcAOXd

>clementine0.9_015716m scaffold_52:50582..53736 -

CTTTAATCCTGACGTCGATAATTACGAAGCCCAATCAAGATCGATCCCCG

AGCACCGAATTAATAAATTACCAAAATTGCCTATTTCTTTGTCTGCTAGT

CTGTACTTTTGAGCAGCGTTTTGACTGACCGAAGAAACAAAAAACAAAGA

AAATGAATCAATTAGTAGCGATGTCGGTGATGCGAGGGCTGATTAACGGC

GGGAAGCACAGTATCGGCTACGCAAGGACGGTGGTGAGATGTCATCCGAA

CGTTTGGGACGGAGATGACATGCCGTTATTGGGGTTGAGAATGATGGTGA

TGATGAGTAGTTATTCTTCTTCTTCGGAATCGGTGCCTGAGAAAGTGAAG

GAGAAAGGAGAGAACGGAATTGTACCGTCGAGTTATTGGGGTATTTCGAG

GCCAAAGATCACTAGAGAGGATGGCAGTCCATGGCCTTGGAATTGTTTCA

TGGTAAAACTATCACCATCACCATCTTAACTTTTGTAAACATTTGTGAAT

TTCTTGACTTGATTTTTTTTTCTTTTGGTTTAATATTTTGTGTAGCCTTG

GGAAACTTATCGGGCAGATTTATCAATTGATTTGAAGAAGCACCATGTGC

CCACAACCTTCCTTGACAAAGTTGCTTACCGGACGGTCAAACTCCTCCGA

ATTCCCACTGATTTGTTTTTTCAGGTATGCCTTTCTTTTCTTGTTATGCT

TTTTCCTAATGAAGTGTTTGGCAAAATTATTAACGTTAATAGTGTTTGGT

AAATAATATGATGATAATTTTGATAAAAATAGCAAAAGATATAGTAACTT

TATCATGAAATAGCTACGGGATAAAATTGATATAGCTTCCAAACACAGCT

GGAAGTATATACGAAAAGAGCCATCTTTTTTTTTTTGAATTATTGTTCTG

ATAAGCCAATTTAGATGCTTGATTTTCCATAATTCAATTATTTCTATGGA

GCACTTTTATTTGTACTGCCAATTAATTGACTTTGAGAATGTGATGTATT

TTCTTCAATACAGAGACGATATGGATGTCGTGCAATGATGCTGGAAACAG

TGGCGGCTGTACCTGGAATGGTTGGAGGAATGTTGCTACACCTCAAGTCT

CTGCGTAAGTTCCAGCATAGTGGTGGTTGGATCAAAGCTCTGCTTGAAGA

AGCAGAGAATGAGAGGATGCATCTGATGACCATGGTGGAGCTTGTGAAGC

CCAAATGGTATGAGAGGATGCTTGTTCTGACTGTGCAGGGTGTCTTTTTC

AATGCATTCTTTGTGCTTTATCTACTCTCACCTAAACTGGCTCATAGAGT

TGTTGGCTACTTGGAGGAGGAGGCTATACACTCTTACACTGAATATCTCA

AGGATATTGATAGTGGATCTATTGAAAATGTTCCAGCCCCTGCTATTGCT

ATTGACTATTGGAGGTTACCTAAGGATGCTACACTCAAGGATGTTATTAC

TGTTATTCGTGCTGACGAAGCTCATCATCGGGATGTCAACCATTTTGCTT

CTGTGAGTATCCCTGTGCGATATATGTTGATATGCCTAACGAATTGTTCT

ATCATTTGCTGCGATAAATCTACCCTGTTATATCCTACTTAAATTTTATT

CCCTTTACTCGTCTTGGGATGTATGAAATTGGTTGCAATAGAAAAATGAT

TATGGATTGTAACAACATTCCAGGCCCTGCCTGATCATGGAAAAAATTTT

AATCCAACAGTTGCTTATTGTGTAAAATGGGGAAGCACAATTAGGCTTTG

CATATTTATACCTAGCTATAACCAATTGCCAAGAGAGGAAACGAGGATCT

GACTGTGTGACCGGGAAAGTAACTTAAAATGATTGCCATGATTTTGTGAG

AACACATACTTGTGCTTTTTGCTTATTTTGATGTGAGAATTGTGTTCTTG

CCTTGCAGGATATACAGTTTCAGGGGAAGGAATTAAGAGATGCTCCTGCC

CCTCTTGGTTACCACTGAGATGGGATGAATTTGAGATTGTTTGTCAGATT

TGTGGTTGAGGTACTTCTACATGGATTGGTCTGAGATAATGAGGTTTCTG

TATATGCTGAGAATAGTACACTAGGAGTATGAAATCTCAAGCTAAATGAA

AAAGGGGTATAGAGTTTTCTGTATATGCTGAGAATAGTACACTAGGAGTA

TGAAGTGTCAAGCTAAATGAAAAAGGGGTATAGACTAAATAAACACATCT

GACTTTCTAAACCCAATTTGGTTGAATTTTATAAGCTTTGAGATTAGACT

GCTTTTGTTTCTGTATGATAAGCAAGTAGCATGTTCCCAGTAGCTCCTCA

GTACATCACCATTTATTTATTTTACTTGTGCTAGCATTTTCTATTACACA

TTCATATGAAGGAGACAGGGAGATGGAGACATCTTGAGTGGAAGGGAATC

GTTGCTTCATAGATGATATTACTGCAAGTTTCTCCAAAAGCAGCATGCAA

AAGGTCAGGATTTCTTCTTGAACTCCCAATATGTTCCCTGATATGAGTGA

AGTGTTGTTGGTGAGAACCCCCAATTGTGCTTTATCCATGTAGTCATAAA

AGAAATCGAAGTGTGGTGCTGGTAGCTCAAAAGGTTGTAAGTTAAGATCT

CTCATAGTCATATGACGTACCAATTCTGTATAATGATGAGGCTGGCAGCT

CTATAATTTTACTGCAAACATATATTATATAGGTGCTTTAATGTAACTGA

AGATATATATGACTGATCGCTAGCATGAGCAAAAGCTATTAAATTAAATG

ATTAGATAAACAAAAATAAGTAATCTTATTTGAAATGTGTTTATGGTGGC

AGTGGATGGAGATGGTACTGTCAGCGAATACACTATTATTATGTGTTTAA

CTTTGGGATTGTGTATCTTTGCTAGAAAGTTCTTCTTGATGGTGAGTTAA

ATGAGAAAATGGTTAGAAGGGAAATTTTTTTTATTCCCGCCTTGTTAGAA

AATGCTGCCTTGTTAGAAAATGCTCTCTGTTGGTAACTTAAAAATAAAAA

GCTCACTGTTTGGAAATGACATTAAGGACCTCATGCAAGATCTTCCAAGT

TTCAAGTTTCAACCAATTAGTATGCAAATCGCTTTATGCCTAGGATTGAA

TAAATAAAAAATAAAAAAGATAAAACGTATCGTCGTTACTTCTGAAGAAG

CATGT

CsAOXa

>orange1.1g018864m scaffold00007:2160731..2164706 -

GGAATATGCTGCCTCCCAAAACCGCCTGTGTGGTCAACCGAACAGCGACA

AAACAAAGCACTTGCTCTTCGATCTTTCCATCCTTCGTCTTGGTGGGAAA

GAATTGGTAACAGAAATGATGACAACTCTCTCTCAAACAGTATCGGTCTT

TTCCAATAAAGCAATAAATCTTAAAAGCGTAGCTTCTTTCAACACCATCA

AGACTTTGCGTTTCAATCCTCCCTCTTCACCTCGATCGCTATCCAGGTTA

GTTTTCAAACGATTATCTCTAATAAATTTTTTTGAACTTAACTTATGAGT

GAATTTGTTTTCTGTAACTTTGTTTCAGGAATTTTTGTCGAGTTCAAGCC

ACAATTTTGCAAGATGAAGAGGAGAAAGTGGTGGTAGAGGAGTCATCTCC

ACTCAAGAATTTTCCAAATGATGATGAGCCACCCGAGACTGGGTCAGCTA

GTGCATTGGAAAAATGGGTTATCAAGTTTGAACAATCTGTTAATATCTTA

CTTACGGTAAATCAAGTGAAGTCTTTTGTAGCTTAAGTGATGCGGTGCTT

GGTGTTGCAAGATTATTAACTCTATTAATCATTTCAGGAATCTGTGATAA

TGGTACTTGATGCCTTGTATCGTGACCGGGATTATGCAAGGTTCTTTGTA

CTGGAGACTATTGCTAGAGTTCCTTATTTTGGTGAGTGTTGTTCTTTCTT

TACATTTTTACCCTTATTTGGTTTCGACGGCATAATTCATCTGGCATAAT

CCATCATTTAGCATTTGTTGTTTGAAGGTGATTTAGGGCCCGTTTGGTGT

TTGGTATCGAGGTGGCTTTTTTTTTTTTTTGTGGGGGGGGGTGTGTGGAG

AGGGAGCTGTGATTGTGAAATAATAGTTGATAGTGTGTGACTGTGTGTGT

GTATGACTTTCAAATAATAATATTAACAACAATAAGAAAAGATTAATTTT

TCATCTTACAAGTGTAGGATTAAATCAACCTAACTTTCAAATTACAGAGA

TAGGTATTTACCCAAATACGTTAGTATTGTTGGCTTTTAACCAATAGTTG

CCCAACGATAATACCAAACTGGGCCTTAATTTGATATAGCATCTTGGTAA

GTGGGGGGCCCTTTAAATGTTATTTCAGTTTTACTCGCTTATTAAAGGAT

TTTTCTTCTGTGGTCATGCCGCTATTAGTTGTGACTTATGGTTTCTACTG

CAGCATTTATTTCTGTTCTGCACATGTATGAGAGTTTTGGTTGGTGGAGA

AGAGCAGATTACCTGAAAGTTCATTTTGCTGAGAGCTGGAATGAGATGCA

TCACTTGCTCATAATGGAAGTAAGGCCCTGTTATTAGATGTTTGTAATAC

TTTCATGTTGTACTCTTATAAAACAGGATAGCTGAGAGGCTTAACCTGTT

ACTTATGGCAGGAAGCCTATTACTGTATCTTGTCATGTTAATTTCTTTAC

TATTTCCTGTTCTCTGATGCATATCTTGGTTGAAGTTTTCTTAATCCAAC

TAGCAGGAAATCTAACATCTGTGAACTAAAGGCAACGACTTAAGACTAGA

GTGGATTGAGGAAAATGGATAGCACCTAATGAGACACTCATTGGAGGAAT

TTTAAACTACGTTGCTCCTCCTTATCGTACCCCCCAAAAAAAAAAATTAT

GTTTCTCTGCTTAAGTTGCAAAAGATATCACTAATAGTTTTCTGTCTCTT

AATCTGATCCAACCACCTTGCCTATTCAGGTCCAAAATAATCTGAAATAT

TTAGACATTACTTGCACATTTGTTTTGTTTCTTAGTCATTTAGTCATTTG

GTTGCAGGAATTGGGAGGAAATGCTTGGTGGTTCGATCGCTTTCTTGCGC

AACATATAGCCGTTGCTTATTATTTTGTGACAGTCTTCATGTATGTAATA

AGCCCAAGAATGGCATGTAAGTATTTGCATTTGGATTTTTAATATTAAAG

TAGAACCTCGTTAGATTAAAATTCAATAAATTTATAACGTTGATTAAATA

ATAATTTTTGTTCGTCTTGATTCTGGACCAATGTAGTAAATCAATAAACA

ATAAATTTATTACATAATAAATTTTAAAAAAACTTATTTTTCTATAGGGC

CCAATTTATACATAAATTAATTATTAACTAATTTGAAACTAAACACATAT

GATAGGTTTATGATTTTATTTTTTTTGGGTACTAGCATAATGCTTTACGC

ATTTCATCTGTCATTGTTGTTTTCTCTACACTTTTCAAATAAAAAGTCAT

TTTTGTATTTTATGTGCTATGTTAAGAGAAATAAGAGAGAGACTAACTTA

ATATATTGCCTCAAAAAAGTCTTCTTTGATAAGATACTATATAACTGTGA

TCCAGTCATGCTATCCAACCAAAGGATTATCAAGTGACTTATTTGCTCAA

TCTGGATCAAATCGAAATCGTATCATGCAACTTGACAGAATCACTGCATT

CTCTAATTATACTATCGTTGTACTTTATAAAAATTCTAATTTTGAAAGTA

ATATTATCTTTTAATAATTATGAATTTATCGATATATTAATATTACTTAA

ATTAATAGATTTTTCATGGTCCCGCAAGGCTATTAATTTATCGAGGTTTT

ACCATATATTCCTAGATTTTCTCATGCTTACTAGAATCTTGTTATTTTAG

TCATCCCTGAATGTTTATAAATCTTTTTCCTTCATTTGATGCTTTTCCCA

TGCAATGCTCCAAGGTATTTTTGAATTTCAAATAGAACAAGTTAGTTTAA

GAACAAGCTAGTATGTTTATGTTTCATCCAACTGCAGATCAATTTTAATG

TATTCATTTTATTGCTTGTGCAGATCACTTTTCGGAATGTGTGGAGAGCC

ATGCTTTTGAAACTTATGACAAATTTATCAAGGCCCAAGGAGGTAAATTT

TTTTTAATGTTTTGGAAAGCTTAGTTTGATTGTCTTGCGCATATACTTCT

ATTTGACATTGAATTGAAGCAAAATAGTTAAATGATTACTATCTTGCTTA

CAGTTCTCATGGTACATTTTTTGGCCCTGATAATGCCACCAAGCAAGTTC

AATTCTCATATAACACTGGCTATGTAAATCTGATGACCTCTACTCTGAGG

ATGCTAGTTAATCGGCCGTTAATTTTTTCAAATGCAAACGGATAATCGGT

GTCTTTTAAGTATGGATTAGTGTGATCAAATGAACTTCATATTCTGCATG

TGTAGTACAGTCCATTTTCACTAGGTTTCACCTGCTCTGTCCCCTCTTTT

TACAGAGAAGTTGAAAAAGATGCCTGCGCCGGCGGTTGCCATTAAGTACT

ATACTGGAGGCGACTTGTACTTATTTGGTAAGTTTGTCTTCTTGCAACCT

TTATAACTGTCATGTAAAACAAGATGTAGCTTTTAACTGAGCCATCTTCA

TTTCTACCCAGATGAATTTCAAACTGCAAGACTCCCAAATTCTCGAAGAC

CAAAAATAGGTAAAGAACGTGTACTGTTTCTAGCACTGCCTATAAGAAGC

TACTCAAGTTCTATACAACAATACTAAATTGCAACTTGCCAGACATACAT

TATGAATACATATTGACGGAAATTGAAATATCTTTCTGATTTGTTTGTTT

TCAGAGAATTTGTATGATGTGTTTTTGAACATTAGAGATGATGAAGCTGA

ACATTGTAAGACGATGAAGGCTTGTCAGACACATGGAAACCTTCGGTCAC

CTCATTCTTATTCAGAGGATGATTTTGGTTGCGAAGATGAGTCTGGCTGC

ATAGTTGCTCAAGCAGATTGTGAAGGAATTGTAGATTGTATAAAGAAAGC

TGTAGCTGTTAGTACTCCCCGAGTAAAGCAAAACTAATCTTTTCTCTTTT

TCTTTATTTTAGAAGGAAGAATGTGATGAAAATTGGCTTAGAATTTACCA

ACAGTTACAGGGCTGTGTTGTATACACAAAAAGAAACCATATAGATCCAT

ACAAAGTTAATCTACACGGGTTTTCT

CsAOXa*

>orange1.1g022654m scaffold00007:2160731..2164706 -

GGAATATGCTGCCTCCCAAAACCGCCTGTGTGGTCAACCGAACAGCGACA

AAACAAAGCACTTGCTCTTCGATCTTTCCATCCTTCGTCTTGGTGGGAAA

GAATTGGTAACAGAAATGATGACAACTCTCTCTCAAACAGTATCGGTCTT

TTCCAATAAAGCAATAAATCTTAAAAGCGTAGCTTCTTTCAACACCATCA

AGACTTTGCGTTTCAATCCTCCCTCTTCACCTCGATCGCTATCCAGGTTA

GTTTTCAAACGATTATCTCTAATAAATTTTTTTGAACTTAACTTATGAGT

GAATTTGTTTTCTGTAACTTTGTTTCAGGAATTTTTGTCGAGTTCAAGCC

ACAATTTTGCAAGATGAAGAGGAGAAAGTGGTGGTAGAGGAGTCATCTCC

ACTCAAGAATTTTCCAAATGATGATGAGCCACCCGAGACTGGGTCAGCTA

GTGCATTGGAAAAATGGGTTATCAAGTTTGAACAATCTGTTAATATCTTA

CTTACGGTAAATCAAGTGAAGTCTTTTGTAGCTTAAGTGATGCGGTGCTT

GGTGTTGCAAGATTATTAACTCTATTAATCATTTCAGGAATCTGTGATAA

TGGTACTTGATGCCTTGTATCGTGACCGGGATTATGCAAGGTTCTTTGTA

CTGGAGACTATTGCTAGAGTTCCTTATTTTGGTGAGTGTTGTTCTTTCTT

TACATTTTTACCCTTATTTGGTTTCGACGGCATAATTCATCTGGCATAAT

CCATCATTTAGCATTTGTTGTTTGAAGGTGATTTAGGGCCCGTTTGGTGT

TTGGTATCGAGGTGGCTTTTTTTTTTTTTTGTGGGGGGGGGTGTGTGGAG

AGGGAGCTGTGATTGTGAAATAATAGTTGATAGTGTGTGACTGTGTGTGT

GTATGACTTTCAAATAATAATATTAACAACAATAAGAAAAGATTAATTTT

TCATCTTACAAGTGTAGGATTAAATCAACCTAACTTTCAAATTACAGAGA

TAGGTATTTACCCAAATACGTTAGTATTGTTGGCTTTTAACCAATAGTTG

CCCAACGATAATACCAAACTGGGCCTTAATTTGATATAGCATCTTGGTAA

GTGGGGGGCCCTTTAAATGTTATTTCAGTTTTACTCGCTTATTAAAGGAT

TTTTCTTCTGTGGTCATGCCGCTATTAGTTGTGACTTATGGTTTCTACTG

CAGCATTTATTTCTGTTCTGCACATGTATGAGAGTTTTGGTTGGTGGAGA

AGAGCAGATTACCTGAAAGTTCATTTTGCTGAGAGCTGGAATGAGATGCA

TCACTTGCTCATAATGGAAGTAAGGCCCTGTTATTAGATGTTTGTAATAC

TTTCATGTTGTACTCTTATAAAACAGGATAGCTGAGAGGCTTAACCTGTT

ACTTATGGCAGGAAGCCTATTACTGTATCTTGTCATGTTAATTTCTTTAC

TATTTCCTGTTCTCTGATGCATATCTTGGTTGAAGTTTTCTTAATCCAAC

TAGCAGGAAATCTAACATCTGTGAACTAAAGGCAACGACTTAAGACTAGA

GTGGATTGAGGAAAATGGATAGCACCTAATGAGACACTCATTGGAGGAAT

TTTAAACTACGTTGCTCCTCCTTATCGTACCCCCCAAAAAAAAAAATTAT

GTTTCTCTGCTTAAGTTGCAAAAGATATCACTAATAGTTTTCTGTCTCTT

AATCTGATCCAACCACCTTGCCTATTCAGGTCCAAAATAATCTGAAATAT

TTAGACATTACTTGCACATTTGTTTTGTTTCTTAGTCATTTAGTCATTTG

GTTGCAGGAATTGGGAGGAAATGCTTGGTGGTTCGATCGCTTTCTTGCGC

AACATATAGCCGTTGCTTATTATTTTGTGACAGTCTTCATGTATGTAATA

AGCCCAAGAATGGCATGTAAGTATTTGCATTTGGATTTTTAATATTAAAG

TAGAACCTCGTTAGATTAAAATTCAATAAATTTATAACGTTGATTAAATA

ATAATTTTTGTTCGTCTTGATTCTGGACCAATGTAGTAAATCAATAAACA

ATAAATTTATTACATAATAAATTTTAAAAAAACTTATTTTTCTATAGGGC

CCAATTTATACATAAATTAATTATTAACTAATTTGAAACTAAACACATAT

GATAGGTTTATGATTTTATTTTTTTTGGGTACTAGCATAATGCTTTACGC

ATTTCATCTGTCATTGTTGTTTTCTCTACACTTTTCAAATAAAAAGTCAT

TTTTGTATTTTATGTGCTATGTTAAGAGAAATAAGAGAGAGACTAACTTA

ATATATTGCCTCAAAAAAGTCTTCTTTGATAAGATACTATATAACTGTGA

TCCAGTCATGCTATCCAACCAAAGGATTATCAAGTGACTTATTTGCTCAA

TCTGGATCAAATCGAAATCGTATCATGCAACTTGACAGAATCACTGCATT

CTCTAATTATACTATCGTTGTACTTTATAAAAATTCTAATTTTGAAAGTA

ATATTATCTTTTAATAATTATGAATTTATCGATATATTAATATTACTTAA

ATTAATAGATTTTTCATGGTCCCGCAAGGCTATTAATTTATCGAGGTTTT

ACCATATATTCCTAGATTTTCTCATGCTTACTAGAATCTTGTTATTTTAG

TCATCCCTGAATGTTTATAAATCTTTTTCCTTCATTTGATGCTTTTCCCA

TGCAATGCTCCAAGGTATTTTTGAATTTCAAATAGAACAAGTTAGTTTAA

GAACAAGCTAGTATGTTTATGTTTCATCCAACTGCAGATCAATTTTAATG

TATTCATTTTATTGCTTGTGCAGATCACTTTTCGGAATGTGTGGAGAGCC

ATGCTTTTGAAACTTATGACAAATTTATCAAGGCCCAAGGAGGTAAATTT

TTTTTAATGTTTTGGAAAGCTTAGTTTGATTGTCTTGCGCATATACTTCT

ATTTGACATTGAATTGAAGCAAAATAGTTAAATGATTACTATCTTGCTTA

CAGTTCTCATGGTACATTTTTTGGCCCTGATAATGCCACCAAGCAAGTTC

AATTCTCATATAACACTGGCTATGTAAATCTGATGACCTCTACTCTGAGG

ATGCTAGTTAATCGGCCGTTAATTTTTTCAAATGCAAACGGATAATCGGT

GTCTTTTAAGTATGGATTAGTGTGATCAAATGAACTTCATATTCTGCATG

TGTAGTACAGTCCATTTTCACTAGGTTTCACCTGCTCTGTCCCCTCTTTT

TACAGAGAAGTTGAAAAAGATGCCTGCGCCGGCGGTTGCCATTAAGTACT

ATACTGGAGGCGACTTGTACTTATTTGGTAAGTTTGTCTTCTTGCAACCT

TTATAACTGTCATGTAAAACAAGATGTAGCTTTTAACTGAGCCATCTTCA

TTTCTACCCAGATGAATTTCAAACTGCAAGACTCCCAAATTCTCGAAGAC

CAAAAATAGGTAAAGAACGTGTACTGTTTCTAGCACTGCCTATAAGAAGC

TACTCAAGTTCTATACAACAATACTAAATTGCAACTTGCCAGACATACAT

TATGAATACATATTGACGGAAATTGAAATATCTTTCTGATTTGTTTGTTT

TCAGAGAATTTGTATGATGTGTTTTTGAACATTAGAGATGATGAAGCTGA

ACATTGTAAGACGATGAAGGCTTGTCAGACACATGGAAACCTTCGGTCAC

CTCATTCTTATTCAGAGGATGATTTTGGTTGCGAAGATGAGTCTGGCTGC

ATAGTTGCTCAAGCAGATTGTGAAGGAATTGTAGATTGTATAAAGAAAGC

TGTAGCTGTTAGTACTCCCCGAGTAAAGCAAAACTAATCTTTTCTCTTTT

TCTTTATTTTAGAAGGAAGAATGTGATGAAAATTGGCTTAGAATTTACCA

ACAGTTACAGGGCTGTGTTGTATACACAAAAAGAAACCATATAGATCCAT

ACAAAGTTAATCTACACGGGTTTTCT

CsAOXb

>orange1.1g037339m scaffold00149:78614..80216 +

ATGTGGCAAGCAGCTGGGGCTAATTATTCCTCCTCTTCATTAACCTCGGG

GAGGCATCTGATGAGTCGTTATCCCGCGGGGATAGTGCGGTATTGGAGTA

GTGCTTCATCATCATCATCATCATCATCATCATCATCATCAGCTCCTCCT

GTAGACTTGCCGAAGGACAAAGAGGAGATCAATCAGCAAGTGTCAAGTGC

AAGCCcTTCCGATCATAAGCAAATGTTAAGAACATTGTTAGCTACTGGGG

CATCATTCCAACTAAGGTTACCAAAGAGGATGGCTCTGCCTGGAGGTGGA

ATTGCTTCAGGGTAAGTGTACATACACATTTACGCAAGCTCACGAGTAAT

AATTAATAATATTACATGTTCTAATCAAGTGGGTAAATTCACTATCAGTT

ACTAGCTAGGGTCTGGTATGAATTCTCAGTTGATCCTCGTGTCATTTGAG

CCATAGCGATGTGATCTACCATTATAATAATTCAAATGATATTGCATTCA

TCGAATAATAACTTGAATGATCGGtGTTATTATTGCAGCCCTGGGAGACC

TAAAAaGCCGACTTGTCAATTGATGTCACCAAGCACCACAAGCCAGAAAA

TtACAGGGACAAATTTGCATACTGGACAGTCCAAGCTCTCAAATTCCCAA

CCCATTTATTCTTCCAGAGACGCCACATGTGTCACGCCATGTTGCTCCAG

ACGGTGGCCGCCGTCCCCGGAATGGTGGGCGGCATGCTCTTGCACTGCAA

GTCACTGAGGAAATTCGAACACAGCGGCGGCTGGATCAAGGCTCTGTTGG

AAGAGGCAGAAAATGAGCGCATGCATTTGATGACATTCATAGAATTGGCC

AGGCCTCAGTGGTACGAGCGAGCCCTGGTGTTTGCCGTACAAGGAGTGTT

TTTTAATGCTTATTTCTTGGCATACTTGGCATCTCCAAAGCTCGCTCACC

GCATTGTGGGTTACTTGGAAGAGGAGGCTGTGAATTCTTACACTGAGTTT

CTTAAAGACTTGGAGAACGGCAGCTTTGAAAATGCTCCGGCTCCGGCTAT

TGCCATTGATTATTGGCGTATGCCTCCTGATTCAACACTTCGTGATGTTG

TGGTGGTCATACGAGCCGATGAGGCGCATCATCGGGACATTAANNNNNNN

NNNNNNNNNNNNNNNNNNNNNNNNNNNNNNNNNNNNNNNNNNNNNNNNNN

NNNNNNNNNNNNNNNNNNNNNNNNNNNNNNNNNNNNNNNNNNNNNNNNNN

NNNNNNNNNNNNNNNNNNNNNNNNNNNNNNNNNNNNNNNNNNNNNNNNNN

NNNNNNNNNNNNNNNNNNNNNNNNNNNNNNNNNNNNNNNNNNNNNNNNNN

NNNNNNNNNNNNNNNNNNNNNNNNNNNNNNNNNNCCACTATGCTTCGGTA

AGAATCGAACTAAAACACACACACACACACACACACAAATTAACGGTTCT

ATTTGTTTGTTTAAATATTTTTAATTGATAATTTTGGTTGTTTGTGTTGC

AGGACATTCAATGTCAAGGGCATGAGCTCAAGGACGCACCTGCTCCGGTT

GGATATCATTGA

CsAOXc

>orange1.1g019765m scaffold00149:82686..85374 +

CTTCCTCCCCAAGAAACAACAAAAGCAGCCAAAACTCTTACCAAATTAAC

AAATCAATAGCAGTCATTTCTTTTCGTCGATCCTTTAATTCTTGAACAAT

GTATCGTGGTGGGGTCCGCTTGTTTTCTACCCTAACCGCACGCACAGCTT

CAACCGAGGCGGCCGCCAGGATCTTGGCCGGCCAAACTAACAGGCATTTA

TCGACCTTCTTGGTTAGGGCTCCGATTCTAGGCTCAAGGAACCGGAGCAC

GTTGGCTTTAGGTGgTGAGAAAGAACAACAGCGGGAGGAGAATGTTCAGA

CGACCGGTGCCGCGGCGgCcGGTGGCAGTGGGAACAAGGATGAGAAGAGG

ATTGTGAGTTACTGGGGCGTGGAGGCTCCTAAAGTTAACAAAGATGATGG

CAGTGAATGGAAATGGAACTGCTTTAGGGTATATGCTCATGAATTGTTAT

TATTAATTTCTTTTCTTGGATCTTTATTTTACGTGTGCTTTAAGCAAATC

ATTGATGTGTCTGTGGTGTTTGTGTTTATATTATTATAGCCATGGGAGGC

TTACGAAGCGGATTTATCAATTGATTTGAAGAAGCACCACGCACCGACGA

CCTTTTCGGATAAAATGGCCTTGTGGACCGTCAAATCACTCAGGTGGCCC

ACTGATTTGTTCTTTCAGGTATATATTTGTTGTTATGTTTTCGATTGATC

AGGGGAGTGGCCGAATAGGGATTTTGGTTCGGGGCAGGGAATTAAATATA

GAAACATGAAACATGAAGGGGTTATTCATAATATCTGTAATTGTAGGGAA

AAaTAAATTGATAAACAATTAAACACTAACCAATTTCTTTTtCTTTTTTT

TTtAGAAACGATTAATAGAAaTATAATTTtGTATTAATAAGGATTTTGGC

TTGATGCTTTAAATaTAAAAATATTATACTTGATTGAGTCTACGACATAA

TATCTGTAAAaTTTAGGGgCCAAATATAAACTTTTCATAGAAATTGACTA

AATTTCAGTTGAATCCCTCAATAATTTCTGAATTTTGGTGAAATTGGTAA

AGATTAATAAAAaCTCTTTTTtATAATTAATTTGAACAGAGAAGATACGG

GTGCCGGGCTATGATGCTCGAAACAGTGGCGGCAGTCCCCGGGATGGTGG

GAGGGATGCTGTTGCATTGCAAGTCATTGAGGCGTTTCGAGCACAGCGGT

GGTTGGATTAAGGCTTTGTTGGAAGAAGCAGAGAATGAACGAATGCATTT

GATGACATTCATGGAGGTAGCAAAGCCTAAATGGTACGAGCGTGCTCTTG

TGTTTGCAGTGCAAGGTGTGTTCTTCAATGCTTACTTCTTGGGCTATCTC

ATcTCGCCTAAgTTCGCTCATCGGATGGTTGGGTACCTTGAGGAAGAAGC

AATCCACTCGTACACCGAGTTCCTCAAGGAGTTGGATAAAGGTAACATTG

AGAACGTTCCTGCTCCGGCCATTGCCACTGATTACTGGAGGTTGCCTCCG

AACTCGACTCTCAAGGATGTTGTGCTGGTTGTAAGAGCAGATGAGGCTCA

CCACCGTGATGTTAATCACTaTGCATCGGTATGATCAGGATTCTCTTTTG

AGCTAAACTATTTGCAGgATACATTTGTTCGGCAGTAACTTTTAATACTT

GCCTTTTTGAAATGCAGGACATACATTACCAAGGGCGCCAATTGAGGGAA

TCTCCAGCTCCACTGGGGTATCACTGATCCAATGATGAAAAGAATCAGAA

TTCAAATACAACATGAATTTTACTACGGTGGTAGTATTGG

CsAOXd

>orange1.1g020532m scaffold00336:129904..133054 +

TTGTGCCATTCACTCGTAGGACTAGTACAATTCAACTCTCCAaTAAGCAG

TtAaTTCAACCAAATTTAAGGGCATAATCGTCTTGTCATTGTCGTCGGTT

AATCAACACCGTTCGCGGTCCAATCATTTCGGAAAAAATTCTCATTACAA

AATTACAAATCCAACTGACCGCTAAAACcTTCTATATCCGCGCGTTGATT

CTTTAATCCTGACGTCGATAATTACGAaGCCCAATCAAGATCGATCCCCG

AGCACCGAATTAATAAATTACCAAAATTGCCTATTTCTTTGTCTGCTAGT

CTGTACTTTTGAGCAGCGTTTTGACTGACCGAAGAAACAAAAAACAAAGA

AAAATGTCGGTGATGCGAGGGCTGATTAACGGCcGGAAGCACAGTATCGG

CTACGCAAGGACGGTGGTGAGATGTCATCCGAACGTTTGGGACGGAGATG

ACATGCCGTTATTGGGGTTGAGAATGATGGTGATGATGAGTAGTTATTCT

TCTTCTTCGGAATCGGTGCCTGAGAAAGTGAAGGAGAAAGGAGAGAACGG

AATTGTACCGTCGAGTTATTGGGGTATTTCGAGGCCAAAGATCACTAGAG

AGGATGGCAGTCCATGGCCTTGGAATTGTTTCATGGTAAAACTATCACCA

TCACCATCTTAACTTTtGTAAACATTTGTGAATTTCTTGACTTGATTTTt

TTTTCTTTTGGTTTAATATTTTGTGTAGCCTTGGGAAACTTATCGGGCAG

ATTTATCAATTGATTTGAAGAAGCACCATGTGCCCACAACCTTCCTTGAC

AAAGTTGCTTACCGGACGGTCAAACTCCTCCGAATTCCCACTGATTTGTT

TTTTCAGGTATGCCTTTCTTTTCTTGTTATGCTTTTTCCTAATGAAGTGT

TTGGCAAAATTATTAACGTTAATAGTGTTTGGTAAATAATATGATGATAA

TTTTGATAAAAATAGCAAAAGATATAGTAACTTTATcATgAAaTAGCTAC

GGGATAAAATtGATATAGCTTCCAAACACAGCTGGAAGTATATACGAAAA

GAGCCATCTTTTTTTTTTtGAATTATTGTTCTGATAAGCCAATTTAGATG

CTTGATTTTCCATAATTCAATTATTTCTATGGAGCACTTTTATTTGTACT

GCCAATTAATTGACTTTGAGAATGTGATGTATTTTCTTCAATACAGAGAC

GaTATGGATGTCGtGCAATGATGCTGGAAACAGTGGCagCTGTACCTGGA

ATGGTTGGAGGAATGTTGCTACACCTCAAGTCTCTGCGTAAGTTCCAGCA

TAGTGGTGGTTGGATCAAAGCTCTGCTTGAAGAAGCAGAGAATGAGAGGA

TGCATCTGATGACCATGGTGGAGCTTGTGAAGCCCAAATGGTATGAGAGG

ATGCTTGTTCTGACTGTGCAGGGTGTCTTTTTCAATGCATTCTTTGTGCT

TTATCTACTCTCACCTAAACTGGCTCATAGAGTTGTTGGCTACTTGGAGG

AGGAGGCTATACACTCTTACACTGAATATCTCAAGGATATTGATAGTGGA

TCTATTGAAAATGTTCCAGCCCCTGCTATTGCTATTGACTATTGGAGGTT

ACCTAAGGATGCTACACTCAAGGATGTTATTACTGTTATTCGTGCTGACG

AAGCtCATCATCGGGATGTCAACCATTTTGCTTCTGTGAGTATCCCTGTG

CGATATATGTTGATATGCCTAACGAATTGTTCTATCATTTGCTGCGATAA

ATCTACCCTGTTATATCCTACTTAAATTTTATTCCCTTTACTCGTCTTGG

GATGTATGAAATTGGTTGCAATAGAAAAATGATTATGGATTGTAACAACA

TTCCAGGCCCTGCCTGATCATGGAAAAAATTTTAATCCAACAGTTGCTTA

TTGTGTAAAATGGGGAAGCACAATTAGGCTTTGCATATTTATACCTAGCT

ATAACCAATTGCCAAGAGAGGAAACGAGGATCTGACTGTGTGACCGGGAA

AGTAACTTAAAATGATTGCCATGATTTTGTGAGAACACATGTGCTTTTTG

CTTATTTTGATGTGAGAATTGTGTTCTTGCCTTGCAGGATATACAGTTTC

AGGGGAAGGAATTAAGAGATGCTCCTGCCCCTCTTGGTTACCACTGAGAT

GGGATGAATTTGAGATTGTTTGTCAGATTTGTGGTTGAGGTACTTCTACA

TGGATTGGTCTGAGATAATGAGGTTTCTGTATATGCTGAGAATAGTACAC

TAGGAGTATGAAATcTCAAGCTAAATGAAAAAGGGGTATAGAGTTTTCTG

TATATGCTGAGAATAGTACACTAGGAGTATGAAGTGTCAAGCTAAATGAA

AAAGGGGTATAGACTAAATAAACACATcTGACTTTCTAAACCCAATTTGG

TTGAATTTTATAAGCTTTGAGATTAGACTGCTTttGTTTCTGTATGATAA

GCAAGTAGCATGTTCCCAGTAGCTCCTCAGTACATCACCATTTATTTATT

TTACTTGTGCTAGCATTTTCTATTAcACATTCATATGAAGGAGACAGGGA

GATGGAGACATCTTGAGTGGAAGGGAATCGTTGCTTCATAGATGATATTa

CTGCAAGTTTCtCCAAAAGCAGCATGCAAAAGGTCAGGATTTCTTCTTGA

ACTCCCAATATGTTCCCTGATATGAGTGAAGTGTTGTTGGTGAGAACCCC

CAATTGTGCTTTATCCATGTAGTCATAAAAGAAATCGAAGTGTGGTGCTG

GTAGCTCAAAAGGTTGTAAGTTAAGATCTCTCATAGTCATATGACGTACC

AATTCTGTATAATGATGAGGCTGGCtGCTCTATAATTTTACTGCAAACAT

ATATTATATAGGTGCTTTAATGTAACTGAAGATATATATGACTGATCGCT

GgCATGAGCAAAAGCTATTAAATTAAATGATTAGATAAACAAAAATAAGT

AATCTTATTTGAAATGTGTTTATGGTGGCAGTGGATGGAGATGGTACTGT

CAGCGAATACACTATTATTATGTGTTTAACTTTGGGATTGTGTATCTTTG

CTAGAAAGTTCTTCTTGATGGTGAGTTAAATGAGAAAATGGTTcGAAGGG

AAaTTTTTTTtATTCCCGCCTTGTTAGAAAATGC
