# Supplementary material for: Alternative oxidase (AOX) constitutes a small family of proteins in Citrus clementina and Citrus sinensis L. Osb
Source: PLoS One. 2017 May 1;12(5):e0176878. doi: 10.1371/journal.pone.0176878 (PMC5411082; doi:10.1371/journal.pone.0176878)
Supplement: S2 Fig — (DOCX) [file pone.0176878.s002.docx]

**S2 Figure. Promotor sequence of the Citrus *AOX* genes (1500 bp upstream excepted for CsAOXd)**

**CcAOXa promotor (1500 bp)**

CTAGACCGGTGGGTTGTTTAAACTCTCTTAATCTTTCGCTCTGATGAATCATAAATAGAAGTTCAATATAATCTAGTAATACTTTATTTTAAAATACGTGTATAATTGGCCTTGCCGTTTTCGCCTTTTGGGGAGCCGGGAGCCCCTTCCAGAATAAGGTGACCCACAACTAGTCAGTTTTTGCCACTTGTTTTTAAACTGGCGATGATGCCGCTTTTTTATTGCCGTTTTCTTTCCCTACCACGAATTTTTTCTACCCCTTTTTGCGTCCGTTTAACATAACTTTTACAAATAAAATTTATTTAAACAGAACTTTTAGTTAAGAGATTTTATACGGAGAATTTTTATTAAAAGTAATTAAATTATTTAGTTGTCGATAAAATTTTTTTTAAAAAATAATAAAACTATTTTAATAGATAATTTTTTAAAAATTATATTATTAAAAAATGAATAAGATTGTAAAATAAATTAAAGACACTTTAGGATAATTTAACTCTTTAATACATTGTACAACTTTTATCTTTAATGATTTTAAATTTAAAATTTTACTAATAAAGACAAAATAACTTATTTCATCTCAAAAATTATACTCAAAATATAACTAAACAATATTAAAATGATTAACAGACTTATAAAACTAAAAAAGTATTGATGACAATCAAATAAATTATATCGGACATGCACTAGTATTCAAAAAAAAAAAAAAAATTGGATGCAACATATTTTAATTGATTTTGGAAAAAAAAATCTATTGTCAAATCTTTACACTTTGCTTAAAAAAAATCCTTTTATAATTGTGATTAAAAGAAAAAATGTAACACGGCACATCAAAATCTTGTAAGTGATTGTCTAAGACGCGATGCGCCGATGTCTACATGGCCACATTTTGAAGGCAACTTTAAAAGGGAACCCTTGATACAATAAATATGCGTGAGCGTGTCCAGATAACCTTCACATGATGCTCGCAAGAATTTCTATCTTCAATTTTCTTGAGAAAACCGTGTGAAGGCTGTTTCTAACTTTCTGTAAAGCTAAATTCACTGTTTAAAAAAAAAAAAAAAAAAAAAACCTTTGAACAAGGCAACAGGCGTGGATAAGAAATTGTTTACAGCTAATTTGGTTGCAGACGTAAGAAAAGGATGACCATTGCAACTGGACAATAATGTGGATGACAGGTCGGTTGAAGTTGACGTCGACAGGAATCCACCGTTCAAGCTTTCCGACTCGGAATAAGCTTCTGTTGTCCACAAAGATTAAATTAAATTAAATTATATTATACGTGAAGCAAGTACATTTAATTTAGACTTTAAATCCATTTTTAATGTTGGTGTGTTGACATCAAGCGATTGCGTCATTGTTGTTCGTCGTCGGGCCATATTCTTGTGGGCTGCCATATCCTCCAGAAATGTAAAACGGTCTATTTAAATGATTCGGCCCAGTCAGGTCATTGGGCTATAAAATGGAGTCATTTGTCGAGCTTTAACAATAGAATGGGCTCAA

**CcAOXb promotor (1500 bp)**

CTTAATTAGAAAAACGCGAAATGTAATTGTTTGTGGGATATGCTTCGACGAAGACCAGCAGTGATGAGCAGACATCTAGCAACAGGGTTCCTCGAGCAGGTATATCTTGTAAGAGTCTAGTAGTCGAGGGAGCAGGAGCAGGAAATATCCTGCTCATCCACGAGCACGTCATCCACGAGGCCAATCTCCTGTTGGAAGCATAAAGCCATTTCTGCTACATTCCTGCTAAGGGTTGAGTCGAGGAGACCCGGTCAACGACAGTTGGCGAGGCATGCTCTCCAGTCCGAGAATCAAGGCACGAAGGCCACACAACCACCCACGACTACGGAAATGGAGCATCCACTTTCGAGAGGTGGGAGGATAACGAGCGCGAATGGAGGGAAGGCCTGAGTTCAGAAGTGACCTATAAAAAGGTAGCGAACGAAGGTAAAAGGGTTAGAATTTTTGACATTATAGCAAAGATAAAGAGGGCTCTAAAGAATTGATACGCTATAAGATTTGTGGCTAGCATTCCCAGGAACACAAAAACCTTCATTTCTGACTTGAACGTCGGAGGGTTTACGCCGGGAAAATAACTGGCGTGCTCTGACCTGTTTCTGTGTACGCAGGAACTTCTGGAGAAAAAGCCTTGCGAGGAGAGATCCTGATCGTGGTAGAGTTGATCGAGCAGAGATCCTGGTCATGTAAGGAGGGTAACTAGAATCTCGCATCAACATTTTGGTGCCGTCTGTGGGGAGCTGAGCAAAAAGCTTCTGTTGACAGCAAGAAGAGGAGTAAAGCAAGTGGAAAAACAATGGAGGCAGGAGGAAGTAGTGCACAAGGGGGTGATACGAGGCTTAACGATTCCGTGACGATGAGGGAGATAGCTAGTGAAGCACGGGAAAAAGCCATGTTTGACCGGATGGAACGGATGGAAAAGCATATGGAGACCCTGACAACCATCCTACATGAGCTGCGGAGTGAACGAAAGGGAATCCAGGAGGAACGGGTGAGAGGTAGTGGAGTGGCACCAGGTCACGATAGTACGAGGAGAAGTCAAACCACCAAGAGATTTGGTGGAGAGAGAGGTAACTTATCTCTGCGGGGAGAAATTCATAGAGAGGAAGATCAATCCCCAGCAAGACAGATTTTTGATGAGGACGACGGGGTGGCAAATGCAGAGGAAACAGAGCTCAGGCAACACTTACATGATGTAGAGCAAGAGCGGGATCAAGTTGCAGCACGTGACCCTGGTCGTGCAGTGCAGCTGGAGGAAGAAGTGCGCAGACTAGCGCAGGTAATTGATGACATGCAAGGAAGGAGCAGAGCTCCTGGTTGGAGGATAATGCTGGACGGAGAATCACCGCTCTCGGCAGAGATCATGAGGGCGGTCATCCCAAGAGATTTTCGCCTCCCAAACCTCAGATATTCGGGGCGAACGGACCCGTTGGTGCACCTAGAGCGCTTCAACGACATAACCGGGGTGCAGGGACTATCTCAAGCCCAAAGATGCAGGGTGTT

**CcAOXc promotor (1500 bp)**

GGGGAGGAAGGCGAAGGGTTTAAGGGAGGGTTTATAAATCTGTTAAGTGATGAAGGTTCGACAAATTGTGTGGACAGGCTGGACCAATAAGATTGGAGGGAAGTTTCCGCACGCTGTTGTTAGAAGAGGCGGCGGGCCGGTTTGGTTATTTTATTTTCTTGGGCAACAAGTACTTTGACGTTTCGTGGGGAACAAGATTCGGTTATCTAAATTTATAGCATTTAATTAGTTGGCGTTTATTTATTTATTTTTTATAAGAACGTGTCTTGCCTTCACTCTGAAACATCTGGATTTCTTACAACTTGATTATTCTACTCAAGTTGTATGATTTTTAATTAGTTACAAAGGTTTAATTGAATTCTTCCGACTATTTATATATGCTATCAATGGTGGCAAGTGGATGCATCAAAATACCAGCATTTGTATTGGTTTGTTTCTCTACGTACACCACTCCCTAAATGTTTTCGTGGTGATCACCAATGAATTCGTCAACAAATGACAGGTGTGTTTAAGGTTAGTATAAAGAGTAATGCTATACGTCCTAAATCTTTCGGCATTCATCAGTTGATTGATGAGGAAAATACAATTAATTCATTTGGAATTTTGAAAAAAAATTTAGAATTTTCAACCAACTAATAAATGACATATCATCTGAGGTAAAATTTAGAATTAAAAAAATTGAGATATCTGTCATTACTCTAGTAGTATAAGTATAAATAGGTGGCCGAAACATTATTATTATTCATTCAGTCACTCTAATCATTGTTTATTTATCCTATTTGCTTTAATATTTATTTATTTTCTTTTTTACTGATTACACTAACAATGGATATCTTCTTTTATATATATATATATATATATTGAGAGCAAATTAATTTATTATTACGAGGAGATTATAATTTAATAGAGGTGCAGTTATGAAAATTATTGTTTTAATTTAGTTATCATTAATGTTGAAGGTAAGTTCATTTGATTTAACATAACGTATATTTTTTCATTATATACATAATTATTACTATATAGAAGTGGATTTAAAAAAATATAATTTATTATAATATAGAGTTTATTTTTATTTATCATTAACTCAAAATGGGATCAAAATTTTTTATTTATGTACAATTATTGCAAGACTCGAACTTATTTATTCCTCTTACTACAGAGAGAAGAATCTTTATAGACAATAGTTAGGTGTTCCCTCATATGTATTAATTCTAATTAATTGGTTGTTAGTGCCAAAGAATTTTTTGTCACATCTATTGTTTTAAAATTCATTCAATATTAGGGAACATCGAGTTGAACGGACACTCACATGAACGGAATTTTCTTGTTCTTTTTTTTCAATTAACTTAGAAATTCCTATTTCATTCAACCAAAACACAAAGTCAGTTACAATAATTAATAGCTGTAATACACAACAAAGATCAATTCATATAATTTTATATACTGATTAAATTAAACAACTGGTCAAACTAATTTCACATTGAAAACTAGACAATAATA

**CcAOXd promotor (1500 bp)**

AATCAACGCGCGGATATAGAAGGTTTTAGCGGTCAGTTGGATTTGTAATTTTGTAATGAGAATTTTTTCCGAAATGATTGGACCGCGAACGGTGTTGATTAACCGACGACAATGACAAGACGATTATGCCCTTAAATTTGGTTGAATTAACTGCTTATTGGAGAGTTGAATTGTACTAGTCCTACGAGTGAATGGCACAACTGGTTGGTTGTGGTTCGCTGCGTCGTGCCTCCATATTACGTCGCCACATAATTATTGACAGGCGGAAACCAAAAACTAATTATTTTGTGGATCCCCTAAGTTGCAGCTCACGTAAGTTACAGCTGCCCGTAAATTTTGATTTAGCAACCTATACTCCATATTATTTATAACTAAAAAATAGAATCGCGCTTAGCACAGTTTTAAAAATATTAAATTAAAAATTTGATAAAATCTAAATTTCCTGAGAAATCATACTGATCTCTTCTCAAAAGGAAGATTAAACAAATTAATTTTGTATGTTTATTTCAATAATAATAAATAGCACATAATAATAATAATAATAATAGTTGACACCTGGCGACGCTGAGACTCATGAGCATTCCACAAAATATAAGTTTCAATTGCATCGAGCCCTCCACCCTTTGCCTTCTGTAAAAGATCTGGCCACATCTGCACAAATACAACGCATATAGAAATAAATCATATCAAAATTTGCATATTTAAAAAATAACAATGCTATTTGATCCAAAAATATTGCCCATGATTTTTCCTAAACATGAAAGCGCACCGTTTTGAATGTGGGATCATGTTTGGCTTGTTAGGAACAAAAAAAATTAAGATAATATTTTCGCAGCAAAGTAGCCTTTTTTTTTTTTTGTTAAAAGAAGAAGAAAATGTGAAAGTGCAAGTGATTTCAGAAATTGTAAATTAATTATTTGCATGCAGTACTTGTGGAGTGCTGCGAGGATATAGTGAATTGCACCAAAGAAGATAACTCGTCGTTCTCCATTAATTATAATGGCAGTTGCATTATAATCGACTTGTGTTGTTGATGATAGCAAGCAAAAAAAAATTAAATTTAGAATAAGAAGAATTAGGCCCTAGTGGCATTGTTGAGGGAAGATGAAATAACTTATCTTCCTATGTTAATTAATTAATGTATTTTAAGCCACTAAAGTTTAATTGCTAGCATAATAAAATTTGAAGAAGTCAAAAGGCACTAAATAAAAGATTGCATAATTAAATATGGGATTATGGCATTAAAATTAGGATTGTTGAAGGAAGATGAAAGAACCTCTCTTCCTATGTTAATTAATTGATGTATTTTAAACCACTAAAGTTTAATTGCTAGCATAATAAAATAAATGAATATTTCAAAAGACTTAATTAATTATAAATAGAAAGAGGAAGAGACATTAAAATTATTAAAATTAAACTTAGACCCTAGTGGCATTATTGAGGGAAGATGAAAGAACCTCTCTTCCTTCCTATATTAATTAATTGATGTATTTTAAGCCA

**CsAOXa/CsAOXa* promotor (1500 bp)**

CTCGTTTGAGCCCATTCTATTGTTAAGCTCGACAAATGACTCCATTTTATAGCCCAATGACCTGACTCGGCCGAATCATTTAAATGGACCGTTTCACATTTCTGGAGGATAATGCAGCCCACAAGAATATGGCCCAACGACGAACAACAATCAATCGCTTGATGTCAACACACCAACATTAAAACTGGATTTAAAGTCATAAATTAAATGTACTTGCTTCACGTATAATATAATATAATTTAATTTAATCTTTGTGGACAACAGACGCTTATTCCGAGTCGGAAAGCTTGAACGGTGGATTCCTGTCGACGTCAACTTCAACCGACCTGTCATCCACATTATTGTCCAGTTGCAATGGTCATCCTTATCTTACGTCTGCAACCAAATTAGCTGTAAACAATTTCTTATCCATGCCTGTTGCCTTGTTCAAAGGTTTGGTTTTTTTTTTTNTTTTTTTTTTTTAAATTTTAAGCAGTGAATTTAGCTTTATAGAAAGTTAGAAACAGCCGAGCATCATGTGAAGGTTATCTGGACACGCTCGCGCATATTGATTGTATCAAGGGTTCCCTTTTAAAGTTGCCTTCAAAATGTGGCCATGTAGACATCGCGTCTTAGACAATCACTTACAAGATTTTGATGTGGCGTGTTACATTTTTTCTTTTAATCACAATTATAAAAGGATTTTTTTTTCTTTTAATCACAATTACATTTTTTCTTTTTTTTTCCAAAATCAATTAAAATACGTTGTATCCATTTTTTTTTTTTGAATACTAGTGCATGTCCGATATAATTTATTTGATTGTCATCAATATTTTTTTAGTTTTATAAGTATGTTAATCATTTTAATATTGTTTAGTTATATTTTGAGTATAATTTTTGAGACGAAATAAGTTATTTTGTCTTTATTAATAAAATTTAAAATTTAAAATCATTAAAGATAAAAGTTGTACAATGTATTAAAGAGTTAAATTGTCTTAAAGTGTTCTTAATTTATTTTACAATCTTATTCATTCTTTTAATAATATAATTTTAAAAAAAAATATCTATTAAAATAATTTTATTATTTTTAATAAAAAAATTATCGACAACTAAATAATTTAATTACTTTTAATAAAAATTCTCCGTATAAAATCTCTTAACTAAAAGTTCTGTTTAAATAAATTTTATTTGTAAAAGTTATGCTAAACGGACGCGAAAAGGGGTAAAAAAAATTCGTGGTAGGGAAAGAAAACGGCAATAAAAAAGCGGCATCATCGCCAGTTTAAAAACAAGTGGCAAAAACTGACTAGTTGTGGGTCACCTTATTCTTGAAGGGGGTCCCCAAAAGGCGAAAACGGCAATGCCAATTATACACGTATTTTAAAATAAAAAGTATTACTAGATCATATTGAACTTCTATTTATGATTCATCGGAGCGAAAGATTAAGAG

**CsAOXb promotor (1500 bp)**

GGGCTTGAGATAGTCCCTGcACCCCGGTTATGTCGTTGAAGCGCTCTAGGTGCACCAACGGGTCCGTTCGCCCCGAATATCTGAGGTTTGGGAGGCGAAAATCTCTTGGGATGACCGCCCTCATGATCTCTGCCGAGAGCGGTGATTCTCCTCCAGCATTATCCTCCAACCAGGAGCTCTGCTCCTTCCTTGCATGTCATCAATTACCTGCGCTAGTCTGCGCACTTCTTCCTCCAGCTGCACTGCACGACCAGGGTCACGTGCTGCAACTTGATCCCGCTCTTGCTCTACATCATGTAAGTGTTGCCTGAGCTCTGTTTCCTCTGCATTTGCCACCCCGTCGTCCTCATCAAAAATCTGTCTTGCTGGGGATTGATCTTCCTCTCTATGAATTTCTCCCCGCAGAGATAAGTTACCTCTCTCTCCACCAAATCTCTTGGTGGTTTGACTTCTCCTCGTACTATCGTGACCTGGTGCCACTCCACTACCTCTCACCCGTTCCTCCTGGATTCCCTTTCGTTCACTCCGCAGCTCATGTAGGATGGTTGTCAGGGTCTCCATATGCTTTTCCATCCGTTCCATCCGGTCAAACATGGCTTTTTCCCGTGCTTCACTAGCTATCTCCCTCATCGTCACGGAATCGTTAAGCCTCGTATCACCCCCTTGTGCACTACTTCCTCCTGCCTCCATTGTTTTTCCACTTGCTTTACTCCTCTTCTTGCTGTCAACAGAAGCTTTTTGCTCAGCTCCCCACAGACGGCACCAAAATGTTGATGCGAGATTCTAGTTACCCTCCTTACATGACCAGGATCTCTGCTCGATCAACTCTACCACGATCAGGATCTCTCCTCGCAAGGCTTTTTCTCCAGAAGTTCCTGCGTACACAGAAACAGGTCAGAGCACGCCAGTTATTTTCCCGGCGTAAACCCTCCGACGTTCAAGTCAGAAATGAAGGTTTTTGTGTTCCTGGGAATGCTAGCCACAAATCTTATAGCGTATCAATTCTTTAGAGCCCTCTTTATCTTTGCTATAATGTCAAAAATTCTAACCCTTTTACCTTCGTTCGCTACCTTTTTATAGGTCACTTCTGAACTCAGGCCTTCCCTCCATTCGCGCTCGTTATCCTCCCACCTCTCGAAAGTGGATGCTCCATTTCCGTAGTCGTGGGTGGTTGTGTGGCCTTCGTGCCTTGATTCTCGGACTGGAGAGCATGCCTCGCCAACTGTCGTTGACCGGGTCTCCTCGACTCAACCCTTAGCAGGAATGTAGCAGAAATGGCTTTATGCTTCCAACAGGAGATTGGCCTCGTGGATGACGTGCTCGTGGATGAGCAGGATATTTCCTGCTCCTGCTCCCTCGACTACTAGACTCTTACAAGATATACCTGCTCGAGGAACCCTGTTGCTAGATGTCTGCTCATCACTGCTGGTCTTCGTCGAAGCATATCCCACAAACAATTACATTTCGCGTTTTtCTAATTAAGCTGATCAATTCCACAA

**CsAOXc promotor (1500 bp)**

TCCTTTATTGTATTTGTTGCCTAGTTTTCAATGTGAAGTTAGTTTGACCAGTTGTTTAATTTAATCAGTATATAAAATTATATGAATTGATCTTTGTTGTGTATTACAGCTATTAATTATTGTAACTGACTTTGTGTTTTGGTTGAATGAAATAGGAATTTCTAAGTTAATTGAAAAAAAAGAACAAGAAAATTCCGTTCATGTGAGTGTCCGTTCAACTCGATGTTCCCTAATATTGAATGAATTTTAAAACAATAGATGTGACAAAAAATTCTTTGGCACTAACAACCAATTAATTAGAATTAATACATATGAGGGAACACCTAACTATTGTCTATAAAGATTCTTCTCTCTGTAGTAAGAGGAATAAATAAGTTCGAGTCTTGCAATAATTGTACATAAATAAAAAATTTTGATCCCATTTTGAGTTAATGATAAATAAAAATAAACTCTATATTATAATAAATTATATTTTTTTAAATCCACTTCTATATAGTAATAATTATGTATATAATGAAAAAATATACGTTATGTTAAATCAAATGAACTTACCTTCAACATTAATGATAACTAAATTAAAACAATAATTTTCATAACTGCACCTCTATTAAATTATAATCTCCTCGTAATAATAAATTAATTTGCTCTCAATATATATATATATATATATAAAAGAAGATATCCATTGTTAGTGTAATCAGTAAAAAAGAAAATAAATAAATATTAAAGCAAATAGGATAAATAAACAATGATTAGAGTGACTGAATGAATAATAATAATGTTTCGGCCACCTATTTATACTTATACTACTAGAGTAATGACAGATATCTCAATTTTTTTAATTCTAAATTTTACCTCAGATGATATGTCATTTATTAGTTGGTTGAAAATTCTAAATTTTTTTTCAAAATTCCAAATGAATTAATTGTATTTTCCTCATCAATCAACTGATGAATGCCGAAAGATTTAGGACGTATAGCATTACTCTTTATACTAACCTTAAACACACCTGTCATTTGTTGACGAATTCATTGGTGATCACCACGAAAACATTTAGGGAGTGGTGTACGTAGAGAAACAAACCAATACAAATGCTGGTATTTTGATGCATCCACTTGCCACCATTGATAGCATATATAAATAGTCGGAAGAATTCAATTAAACCTTTGTAACTAATTAAAAATCATACAACTTGAGTAGAATAATCAAGTTGTAAGAAATCCAGATGTTTCAGAGTGAAGGCAAGACACGTTCTTATAAAAAATAAATAAATAAACGCCAACTAATTAAATGCTATAAATTTAGATAACCGAATCTTGTTCCCCACGAAACGTCAAAGTACTTGTTGCCCAAGAAAATAAAATAACCAAACCGGCCCGCCGCCTCTTCTAACAACAGCGTGCGGAAACTTCCCTCCAATCTTATTGGTCCAGCCTGTCCACACAATTTGTCGAACCTTCATCACTTAACAGATTTATAAACCCTCCCTTAAACCCTTCG

**CsAOXd promotor (353 bp)**

TTGTGCCATTCACTCGTAGGACTAGTACAATTCAACTCTCCAaTAAGCAGTtAaTTCAACCAAATTTAAGGGCATAATCGTCTTGTCATTGTCGTCGGTTAATCAACACCGTTCGCGGTCCAATCATTTCGGAAAAAATTCTCATTACAAAATTACAAATCCAACTGACCGCTAAAACcTTCTATATCCGCGCGTTGATTCTTTAATCCTGACGTCGATAATTACGAaGCCCAATCAAGATCGATCCCCGAGCACCGAATTAATAAATTACCAAAATTGCCTATTTCTTTGTCTGCTAGTCTGTACTTTTGAGCAGCGTTTTGACTGACCGAAGAAACAAAAAACAAAGAAAA
