# Supplementary material for: Alternative oxidase (AOX) constitutes a small family of proteins in Citrus clementina and Citrus sinensis L. Osb
Source: PLoS One. 2017 May 1;12(5):e0176878. doi: 10.1371/journal.pone.0176878 (PMC5411082; doi:10.1371/journal.pone.0176878)
Supplement: S3 Fig — (DOCX) [file pone.0176878.s003.docx]

**S3 Figure. List of the *cis*-elements found in the promotor regions of the citrus *AOX* genes.** The number of elements by function was used to obtain the Figure 2 (excepted the categories indicated by *)

| ***Citrus clementina*** | | | | | |
| --- | --- | --- | --- | --- | --- |
| **GENE** | **NAME** | **FUNCTION** | **POSITION** | **NUMBER OF ELEMENTS** | **NUMBER OF ELEMENTS BY FUNCTION** |
| *CcAOXa* | AT1-motif | part of a light responsive module | 458 - | 1 | 10 |
|  | GA-motif | part of a light responsive element | 413 + | 1 |  |
|  | GAG-motif | part of a light responsive element | 22 - | 1 |  |
|  | G-Box | cis-acting regulatory element involved in light responsiveness | 95 -, 1277 - | 2 |  |
|  | I-box | part of a light responsive element | 152 +, 1391 - | 2 |  |
|  | TCT-motif | part of a light responsive element | 1129 - | 1 |  |
|  | ATCT-motif | part of a conserved DNA module involved in light responsiveness | 65 +, 1251 - | 2 |  |
|  | HSE | cis-acting element involved in heat stress responsiveness | 244 -, 1098 - , 379 - | 3 | 3 |
|  | MBS | MYB binding site involved in drought-inducibility | 1150 + | 1 | 1 |
|  | TC-rich repeats | cis-acting element involved in defense and stress responsiveness | 227 +, 1129 -, 984 + | 3 | 3 |
|  | ABRE | cis-acting element involved in the abscisic acid responsiveness | 95 +, 1277 + | 2 | 2 |
|  | GARE-motif | gibberellin-responsive element | 305 +, 1237 + | 2 | 3 |
|  | P-box | gibberellin-responsive element | 124 + | 1 |  |
|  | TCA-element | cis-acting element involved in salicylic acid responsiveness | 1131 + | 1 | 1 |
|  | CGTCA-motif | cis-acting regulatory element involved in the MeJA-responsiveness | 1188 -, 1350+ | 2 | 4 |
|  | TGACG-motif | cis-acting regulatory element involved in the MeJA-responsiveness | 1188 +, 1350 - | 2 |  |
|  | O2-site | cis-acting regulatory element involved in zein metabolism regulation | 1391 -jj | 1 | 1 |
|  | CAT-box | cis-acting regulatory element related to meristem expression | 183 + | 1 | 1 |
|  | Skn-1_motif | cis-acting regulatory element required for endosperm expression | 652 -, 1444 +, 1169 -, 1465 +, 1140 -, 1351 + | 6 | 6 |
|  | circadian | cis-acting regulatory element involved in circadian control | 64 + | 1 | 1 |
|  | AAT-box* | common cis-acting element in promoter and enhancer regions | 105 -, 221 - , 708 -, 1447 - | 4 | 4 |
|  | TATA-box* | core promoter element around -30 of transcription start | 67 +, 101 +, 300 +, 328 - , 329 -, 330 -, 331 +, 432 -, 433 +, 585 -, 586 +, 597 +, 630 -, 631 +, 668 - , 669 +, 758 -, 787 -, 788 -, 789 -, 790 -, 1269 -, 1270 -, 1274 -, 1275 -, 1419 +, 1454 + | 27 | 27 |
| *CcAOXb* | CATT-motif | part of a light responsive element | 507 + | 1 | 17 |
|  | G-Box | cis-acting regulatory element involved in light responsiveness | 1220- | 1 |  |
|  | G-box | cis-acting regulatory element involved in light responsiveness | 164+, 1232-, 317, 171, 1220+ | 5 |  |
|  | Sp1 | light responsive element | 312+,1389+,1145-, 354-,1364+ | 5 |  |
|  | TCCC-motif | part of a light responsive element | 856- | 1 |  |
|  | TCT-motif | part of a light responsive element | 108- | 1 |  |
|  | as-2-box | involved in shoot-specific expression and light responsiveness | 1320+ | 1 |  |
|  | box II | part of a light responsive element | 171+ | 1 |  |
|  | GATT-motif | part of a light responsive element | 1307+ | 1 |  |
|  | MBS | MYB Binding Site; MYB binding site involved in drought-inducibility | 248+, 1367+, 572+, 258-, 894- | 5 | 5 |
|  | TC-rich repeats | cis-acting element involved in defense and stress responsiveness | 615- | 1 | 1 |
|  | ARE | cis-acting regulatory element essential for the anaerobic induction | 1037 - | 1 | 1 |
|  | ABRE | cis-acting element involved in the abscisic acid responsiveness | 1220 - | 1 | 1 |
|  | TATC-box | cis-acting element involved in gibberellin-responsiveness | 33- | 1 | 3 |
|  | GARE-motif | gibberellin-responsive element | 751+, 1163+ | 2 |  |
|  | CGTCA-motif | cis-acting regulatory element involved in the MeJA-responsiveness | 166 +,848 - | 2 | 4 |
|  | TGACG-motif | cis-acting regulatory element involved in the MeJA-responsiveness | 166-, 848+ | 2 |  |
|  | TGA-element | auxin-responsive element | 253+, 1447+ | 2 | 2 |
|  | O2-site | cis-acting regulatory element involved in zein metabolism regulation | 163 -, 1282+ | 2 | 2 |
|  | CAT-box | cis-acting regulatory element related to meristem expression | 1002 - | 1 | 2 |
|  | CCGTCC-box | cis-acting regulatory element related to meristem specific activation | 1329 - | 1 |  |
|  | Skn-1_motif | cis-acting regulatory element required for endosperm expression | 167+, 1283-, 678+, 1369+ | 4 | 4 |
|  | circadian | cis-acting regulatory element involved in circadian control | 1207- | 1 | 1 |
|  | Box-W1 | fungal elicitor responsive element | 249-, 893 | 2 | 2 |
|  | A-box* | cis-acting regulatory element | 1329 - | 1 | 1 |
|  | CCAAT-box* | MYBHv1 binding site | 1423 - | 1 | 1 |
|  | W box* |  | 249-,893+ | 2 | 2 |
|  | TATA-box* | core promoter element around -30 of transcription start | 100+, 450-, 490+404+451+402+ | 6 | 6 |
|  | CAAT-box* | common cis-acting element in promoter and enhancer regions | 180+ | 1 | 1 |
|  | plant_AP-2-like* |  | 1228- | 1 | 1 |
| *CcAOXc* | ACE | cis-acting element involved in light responsiveness | 455- | 1 | 18 |
|  | AE-box | part of a module for light response | 430- | 1 |  |
|  | Box 4 | part of a conserved DNA module involved in light responsiveness | 585+, 1214-, 946-, 1394-,870-, 1204- | 6 |  |
|  | G-Box | cis-acting regulatory element involved in light responsiveness | 258- | 1 |  |
|  | G-box | cis-acting regulatory element involved in light responsiveness | 258- | 1 |  |
|  | I-box | part of a light responsive element | 200- | 1 |  |
|  | L-box | part of a light responsive element | 1449+ | 1 |  |
|  | Sp1 | light responsive element | 1-,23- | 2 |  |
|  | Box I | light responsive element | 602- | 1 |  |
|  | TCT-motif | part of a light responsive element | 293+,1148+ | 2 |  |
|  | CATT-motif | part of a light responsive element | 553+ | 1 |  |
|  | HSE | cis-acting element involved in heat stress responsiveness | 608+,1315-, 1097- | 3 | 3 |
|  | MBS | MYB binding site involved in drought-inducibility | 561-,1383-, 912-,1457+ | 4 | 4 |
|  | TC-rich repeats | is-acting element involved in defense and stress responsiveness | 289+,572- | 2 | 2 |
|  | ARE | cis-acting regulatory element essential for the anaerobic induction | 426+ | 1 | 1 |
|  | LTR | cis-acting element involved in low-temperature responsiveness | 548-,724+ | 2 | 2 |
|  | TCA-element | cis-acting element involved in salicylic acid responsiveness | 119+,1160+,307- | 3 | 3 |
|  | CGTCA-motif | cis-acting regulatory element involved in the MeJA-responsiveness | 175-,486+ | 2 | 4 |
|  | TGACG-motif | cis-acting regulatory element involved in the MeJA-responsiveness | 175+,486- | 2 |  |
|  | O2-site | cis-acting regulatory element involved in zein metabolism regulation | 642- | 1 | 1 |
|  | Skn-1_motif | cis-acting regulatory element required for endosperm expression | 495-,489+,639- | 3 | 3 |
|  | HD-Zip 1 | element involved in differentiation of the palisade mesophyll cells | 1116- | 1 | 1 |
|  | HD-Zip 2 | element involved in the control of leaf morphology development | 1116- | 1 | 1 |
|  | Box-W1 | fungal elicitor responsive element | 1462- | 1 | 1 |
|  | ATGCAAAT motif* | cis-acting regulatory element associated to the TGAGTCA motif | 418- | 1 | 1 |
|  | 5UTR Py-rich stretch* | cis-acting element conferring high transcription levels | 1159- | 1 | 1 |
|  | TA-rich region* | enhancer | 838-,842-,840- | 3 | 3 |
|  | CAAT-box* | common cis-acting element in promoter and enhancer regions | 83 +, 91 -, 424-, 476 +,1218 - | 5 | 5 |
|  | TATA-box* | core promoter element around -30 of transcription start | 30-,31-,211-,212-,213+,248-,249-250-,251-252+,368-,370-,371-,372+,374+,518+,533+,705+,836-,837-,838-,839-,840-,842-,844-,846-,848-,850-,852-,854-,856-,893-,894-,985-,997-,998-,1000-,1014-,1016-,1018-,1040-,1049-,1050-,1055-,1063-,1170-,1171-,1172- | 48 | 48 |
|  | W box* |  | 1462- | 1 | 1 |
| *CcAOXd* | Box 4 | part of a conserved DNA module involved in light responsiveness | 486+,1367-,1134-,1477-,989-1473-,1292-,908-,1130- | 9 | 21 |
|  | Box I | light responsive element | 1353+ | 1 |  |
|  | CATT-motif | part of a light responsive element | 577+ | 1 |  |
|  | G-Box | cis-acting regulatory element involved in light responsiveness | 309+ | 1 |  |
|  | G-box | cis-acting regulatory element involved in light responsiveness | 222-,309- | 2 |  |
|  | GA-motif | part of a light responsive element | 470+,1460-,1259+ | 3 |  |
|  | GATA-motif | part of a light responsive element | 817+ | 1 |  |
|  | GT1-motif | light responsive element | 97-, 98- | 2 |  |
|  | Sp1 | light responsive element | 616+ | 1 |  |
|  | MBS | MYB Binding Site; MYB binding site involved in drought-inducibility | 29+,1003-,147+,33-,197+ | 5 | 5 |
|  | TC-rich repeats | cis-acting element involved in defense and stress responsiveness | 867- | 1 | 1 |
|  | ARE | cis-acting regulatory element essential for the anaerobic induction | 266-,1309- | 2 | 2 |
|  | LTR | cis-acting element involved in low-temperature responsiveness | 68+ | 1 | 1 |
|  | ABRE | cis-acting element involved in the abscisic acid responsiveness | 309-, 550+ | 2 | 2 |
|  | P-box | gibberellin-responsive element | 467-,1192-1189- | 3 | 3 |
|  | TGA-element | auxin-responsive element | 979- | 1 | 1 |
|  | ERE | ethylene-responsive element | 1352+ | 1 | 1 |
|  | CAT-box | cis-acting regulatory element related to meristem expression | 1084-,1427-,1149+ | 3 | 3 |
|  | Skn-1_motif | cis-acting regulatory element required for endosperm expression | 111- | 1 | 2 |
|  | GCN4_motif | cis-regulatory element involved in endosperm expression | 792- | 1 |  |
|  | circadian | cis-acting regulatory element involved in circadian control | 599+ | 1 | 1 |
|  | WUN-motif | wound-responsive element | 49-,435+ | 2 | 2 |
|  | AAGAA-motif* |  | 1268+, 1449+ | 2 | 2 |
|  | AT-rich element* | binding site of AT-rich DNA binding protein (ATBP-1) | 670+ | 1 | 1 |
|  | TATA-box* | core promoter element around -30 of transcription start | 14+,350+,364-,365-,366+,590+,669+,698+,949-,994-,995-,1011-,1012-,1372-,1398-,1470- | 16 | 16 |
|  | CAAT-box* | common cis-acting element in promoter and enhancer regions | 76-,156-,734- | 3 | 3 |

| ***Citrus sinensis*** | | | | | |
| --- | --- | --- | --- | --- | --- |
| **GENE** | **NAME** | **FUNCTION** | **POSITION** | **NUMBER OF ELEMENTS** | **NUMBER OF ELEMENTS BY FUNCTION** |
| CsAOXa | 3-AF1 binding site | light responsive element | 1037- | 1 | 14 |
|  | AT1-motif | part of a light responsive module | 988+ | 1 |  |
|  | ATCT-motif | part of a conserved DNA module involved in light responsiveness | 241+ | 1 |  |
|  | Box 4 | part of a conserved DNA module involved in light responsiveness | 904- | 1 |  |
|  | G-Box | cis-acting regulatory element involved in light responsiveness | 219+,1351+ | 2 |  |
|  | G-box | cis-acting regulatory element involved in light responsiveness | 219-,1351- | 2 |  |
|  | GATA-motif | part of a light responsive element | 1299- | 1 |  |
|  | I-box | part of a light responsive element | 360-,1297-,362- | 3 |  |
|  | TCT-motif | part of a light responsive element | 367+ | 1 |  |
|  | as-2-box | involved in shoot-specific expression and light responsiveness | 359- | 1 |  |
|  | HSE | cis-acting element involved in heat stress responsiveness | 394+,1204+,1034+,1205+ | 4 | 4 |
|  | MBS | MYB binding site involved in drought-inducibility | 346- | 1 | 1 |
|  | TC-rich repeats | cis-acting element involved in defense and stress responsiveness | 1222- | 1 | 1 |
|  | ARE | cis-acting regulatory element essential for the anaerobic induction | 435+ | 1 | 1 |
|  | ABRE | cis-acting element involved in the abscisic acid responsiveness | 219-,1351- | 2 | 2 |
|  | GARE-motif | gibberellin-responsive element | 258-,1147- | 2 | 3 |
|  | P-box | gibberellin-responsive element | 1321- | 1 |  |
|  | TCA-element | cis-acting element involved in salicylic acid responsiveness | 750+ | 1 | 1 |
|  | CGTCA-motif | cis-acting regulatory element involved in the MeJA-responsiveness | 309+ | 1 | 2 |
|  | TGACG-motif | cis-acting regulatory element involved in the MeJA-responsiveness | 309- | 1 |  |
|  | AuxRR-core | cis-acting regulatory element involved in auxin responsiveness | 83- | 1 | 2 |
|  | TGA-element | auxin-responsive element | 135+ | 1 |  |
|  | CAT-box | cis-acting regulatory element related to meristem expression | 1270- | 1 | 1 |
|  | Skn-1_motif | cis-acting regulatory element required for endosperm expression | 35-,357+,195+,801+,56-,328+ | 6 | 6 |
|  | circadian | cis-acting regulatory element involved in circadian control | 1380- | 1 | 1 |
|  | TATA-box* | core promoter element around -30 of transcription start | 44-,45-,46-,47+,223+,228+,233+,485-,486-, 487+, 670-, 671+,785-,820-,821-,822-,823-,856-,857-868-, 1022-,1060-,1124+,1151-,1346-,1347- | 26 | 26 |
|  | CAAT-box* | common cis-acting element in promoter and enhancer regions | 53+,1232+,1335+,1342+ | 4 | 4 |
| CsAOXb | Sp1 | light responsive element | 89-,1165-,332+,114-,1123+ | 5 | 18 |
|  | G-Box | cis-acting regulatory element involved in light responsiveness | 257+ | 1 |  |
|  | G-box | cis-acting regulatory element involved in light responsiveness | 245+,1313-,1160-,257+,1302+ | 5 |  |
|  | CATT-motif | part of a light responsive element | 970- | 1 |  |
|  | TCCC-motif | part of a light responsive element | 620+ | 1 |  |
|  | TCT-motif | part of a light responsive element | 1369+ | 1 |  |
|  | TCT-motif | part of a light responsive element | 1369+ | 1 |  |
|  | as-2-box | involved in shoot-specific expression and light responsiveness | 153- | 1 |  |
|  | box II | part of a light responsive element | 1302- | 1 |  |
|  | GATT-motif | part of a light responsive element | 164- | 1 |  |
|  | MBS | MYB Binding Site; MYB binding site involved in drought-inducibility | 111-,1229-,905-,583+,1219+ | 5 | 5 |
|  | TC-rich repeats | cis-acting element involved in defense and stress responsiveness | 858+ | 1 | 1 |
|  | ARE | cis-acting regulatory element essential for the anaerobic induction | 440+ | 1 | 1 |
|  | ABRE | cis-acting element involved in the abscisic acid responsiveness | 257+ | 1 | 1 |
|  | GARE-motif | gibberellin-responsive element | 313-,725- | 2 | 3 |
|  | TATC-box | cis-acting element involved in gibberellin-responsiveness | 1443+ | 1 |  |
|  | CGTCA-motif | cis-acting regulatory element involved in the MeJA-responsiveness | 630+,1312- | 2 | 4 |
|  | TGACG-motif | cis-acting regulatory element involved in the MeJA-responsiveness | 630-,1312+ | 2 |  |
|  | TGA-element | auxin-responsive elemen | 31-,1224- | 2 | 2 |
|  | O2-site | cis-acting regulatory element involved in zein metabolism regulation | 191-,1310+ | 2 | 2 |
|  | CAT-box | cis-acting regulatory element related to meristem expression | 475+ | 1 | 1 |
|  | Skn-1_motif | cis-acting regulatory element required for endosperm expression | 110-,800-,195+,1311- | 4 | 4 |
|  | circadian | cis-acting regulatory element involved in circadian control | 266+ | 1 | 1 |
|  | Box-W1 | fungal elicitor responsive element | 584-,1228+ | 2 | 2 |
|  | CCAAT-box* | MYBHv1 binding site | 55+ | 1 | 1 |
|  | CAAT-box* | common cis-acting element in promoter and enhancer regions | 1298- | 1 | 1 |
|  | TATA-box* | core promoter element around -30 of transcription start | 988-,1072-,1069-,1379-,1028-,1074-,1071-,1073-,989-,1075- | 10 | 10 |
| CsAOXc | ACE | cis-acting element involved in light responsiveness | 1046+ | 1 | 17 |
|  | AE-box | part of a module for light response | 1073+ | 1 |  |
|  | Box 4 | part of a conserved DNA module involved in light responsiveness | 111+,635+,301+,920-,291+,559+ | 6 |  |
|  | Box I | light responsive element | 902+ | 1 |  |
|  | G-Box | cis-acting regulatory element involved in light responsiveness | 1247+ | 1 |  |
|  | G-box | cis-acting regulatory element involved in light responsiveness | 1247+ | 1 |  |
|  | I-box | part of a light responsive element | 1302+ | 1 |  |
|  | L-box | part of a light responsive element | 50- | 1 |  |
|  | Sp1 | light responsive element | 1482+ | 1 |  |
|  | TCT-motif | part of a light responsive element | 357-,1212- | 2 |  |
|  | CATT-motif | part of a light responsive element | 952- | 1 |  |
|  | HSE | cis-acting element involved in heat stress responsiveness | 186+,893-,404+ | 3 | 3 |
|  | MBS | MYB binding site involved in drought-inducibility | 48-,593+,122+,944+ | 4 | 4 |
|  | TC-rich repeats | cis-acting element involved in defense and stress responsiveness | 929+,1212- | 2 | 2 |
|  | ARE | cis-acting regulatory element essential for the anaerobic induction | 1079- | 1 | 1 |
|  | LTR | cis-acting element involved in low-temperature responsiveness | 781-,957+ | 2 | 2 |
|  | TCA-element | cis-acting element involved in salicylic acid responsiveness | 341-,1382-,1194 | 3 | 3 |
|  | CGTCA-motif | cis-acting regulatory element involved in the MeJA-responsiveness | 1020-,1331+ | 2 | 4 |
|  | TGACG-motif | cis-acting regulatory element involved in the MeJA-responsiveness | 1020+,1331- | 2 |  |
|  | O2-site | cis-acting regulatory element involved in zein metabolism regulation | 859+ | 1 | 1 |
|  | Skn-1_motif | cis-acting regulatory element required for endosperm expression | 817-,1011+,867+ | 3 | 3 |
|  | HD-Zip 1 | element involved in differentiation of the palisade mesophyll cells | 386+ | 1 | 1 |
|  | HD-Zip 2 | element involved in the control of leaf morphology development | 386+ | 1 | 1 |
|  | Box-W1 | fungal elicitor responsive element | 43+ | 1 | 1 |
|  | CAAT-box* | common cis-acting element in promoter and enhancer regions | 288+,1030-,1082+,1415+,1423- | 5 | 5 |
|  | 5UTR Py-rich stretch* | cis-acting element conferring high transcription levels | 342+ | 1 | 1 |
|  | TA-rich region* | enhancer | 647+,651+,649+ | 3 | 3 |
|  | TATCCAT/C-motif* |  | 679+ | 1 | 1 |
|  | TATA-box* | core promoter element around -30 of transcription start | 68+,70+,77-,78+,335+,337+,439+,452+,456-,457+,466-,467+,487+,489+,491+,507+,509+,522+,612-,613+,649-,651+,653+,655+,657+,659+,661+,663+,665+,667+,794-,795-,796-,801-,802-,974,-987-,988-,989-,1133+,1253+,1254-,1255+,1471-,1473-,1474-,1475+ | 47 | 47 |
| CsAOXd | G-Box | cis-acting regulatory element involved in light responsiveness | 56+ | 1 | 4 |
|  | AE-box | part of a module for light response | 332 + | 1 |  |
|  | Box 4 | part of a conserved DNA module involved in light responsiveness | 258 - | 1 |  |
|  | GT1-motif | light responsive element | 96 + | 1 |  |
|  | MBS | MYB binding site involved in drought-inducibility | 47 -, 165 -, 161 +, 325 - | 4 | 4 |
|  | LTR | cis-acting element involved in low-temperature responsiveness | 126 - | 1 | 1 |
|  | ABRE | cis-acting element involved in the abscisic acid responsiveness | 56- | 1 | 1 |
|  | TGACG-motif | cis-acting regulatory element involved in the MeJA-responsiveness | 209 + | 1 | 2 |
|  | CGTCA-motif | cis-acting regulatory element involved in the MeJA-responsiveness | 209 - | 1 |  |
|  | Skn-1_motif | cis-acting regulatory element required for endosperm expression | 84 + | 1 | 1 |
|  | WUN-motif | wound-responsive element | 141 +, 218 + | 2 | 2 |
|  | Box 4* |  | 3+ | 1 | 1 |
|  | CAAT-box* | common cis-acting element in promoter and enhancer regions | 208+ | 1 | 1 |
|  | G-box* |  | 56- | 1 | 1 |
|  | TATA-box* | core promoter element around -30 of transcription start | 26-,27+,97+,102+,104+,106+,111-,112-,113- | 10 | 10 |
|  | Unnamed__4* |  | 93+,176+ | 2 | 2 |
