# Supplementary material for: Alternative oxidase (AOX) constitutes a small family of proteins in Citrus clementina and Citrus sinensis L. Osb
Source: PLoS One. 2017 May 1;12(5):e0176878. doi: 10.1371/journal.pone.0176878 (PMC5411082; doi:10.1371/journal.pone.0176878)
Supplement: S4 Fig — (DOCX) [file pone.0176878.s004.docx]

**S4 Figure.** **Amino acid sequences of AOX from *C. clementina* and *C. sinensis*.** The signal peptide is underlined. The AOX-pfam01786 functional domain is indicated in gray. Putative phosphorylation sites are indicated in blue. Putative glycosylation sites are indicated in red. Conserved domains are squared.

>*CcAOXa*

MMTTLSQTVSVFSNKAINLKSVASFNTIKTLRFNPPSSPRSLSRKFCRVQATILQDEEEKVVVEESSPLKNFPNDDEPPETGSASALEKWVIKFEQSVNILLTESVIMVLDALYRDRDYARFFVLETIARVPYFGFISVLHMYESFGWWRRADYLKVHFAESWNEMHHLLIMEELGGNAWWFDRFLAQHIAVAYYFVTVFMYVISPRMACHFSECVESHAFETYDKFIKAQGGKLKKMPAPAVAIKYYTGADLYLFGEFQTARLPNSRRPKIGNLYDVFLNIRDDEAEHCKTMKACQTHGNLRSPHSYSEDDFGCEDESGCIVAQADCEGIVDCIKKAVAVSTPRVKQN

*>CcAOXb*

MSTYRATARILRSLMWQAAGANYSSSSLTSGRHLMSRYPAGIVRYWSSASSSSSSSSSSSSAPPVDLPKDKEEINQQNIVSYWGIIPTKVTKEDGSAWRWNCFRHHKPENYRDKFAYWTVQALKFPTHLFFQRRHMCHAMLLQTVAAVPGMVGGMLLHCKSLRKFEHSGGWIKALLEEAENERMHLMTFIELARPQWYERALVFAVQGVFFNAYFLAYLASPKLAHRIVGYLEEEAVNSYTEFLKDLENGSFENAPAPAIAIDYWRMPPDSTLRDVVVVIRADEAHHRDINHYASDIQCQGHELKDAPA

*>CcAOXc*

MMYRGGVRLFSTLTARTASTEAAARILAGQTNRHLSTFLVRAPILGSRNRSTLALGGEKEQQREENVQTTGAAAAGGSGNKDEKRIVSYWGVEAPKVNKDDGSEWKWNCFRPWEAYEADLSIDLKKHHAPTTFSDKMALWTVKSLRWPTDLFFQRRYGCRAMMLETVAAVPGMVGGMLLHCKSLRRFEHSGGWIKALLEEAENERMHLMTFMEVAKPKWYERALVFAVQGVFFNAYFLGYLISPKFAHRMVGYLEEEAIHSYTEFLKELDKGNIENVPAPAIATDYWRLPPNSTLKDVVLVVRADEAHHRDVNHFASDIHYQGRQLRESPAPLGYH

*>CcAOXd*

MNQLVAMSVMRGLINGGKHSIGYARTVVRCHPNVWDGDDMPLLGLRMMVMMSSYSSSSESVPEKVKEKGENGIVPSSYWGISRPKITREDGSPWPWNCFMPWETYRADLSIDLKKHHVPTTFLDKVAYRTVKLLRIPTDLFFQRRYGCRAMMLETVAAVPGMVGGMLLHLKSLRKFQHSGGWIKALLEEAENERMHLMTMVELVKPKWYERMLVLTVQGVFFNAFFVLYLLSPKLAHRVVGYLEEEAIHSYTEYLKDIDSGSIENVPAPAIAIDYWRLPKDATLKDVITVIRADEAHHRDVNHFASDIQFQGKELRDAPAPLGYH

>CsAOXa

MMTTLSQTVSVFSNKAINLKSVASFNTIKTLRFNPPSSPRSLSRNFCRVQATILQDEEEKVVVEESSPLKNFPNDDEPPETGSASALEKWVIKFEQSVNILLTESVIMVLDALYRDRDYARFFVLETIARVPYFAFISVLHMYESFGWWRRADYLKVHFAESWNEMHHLLIMEELGGNAWWFDRFLAQHIAVAYYFVTVFMYVISPRMAYHFSECVESHAFETYDKFIKAQGEKLKKMPAPAVAIKYYTGGDLYLFDEFQTARLPNSRRPKIENLYDVFLNIRDDEAEHCKTMKACQTHGNLRSPHSYSEDDFGCEDESGCIVAQADCEGIVDCIKKAVAVSTPRVKQN

*>CsAOXa**

MMTTLSQTVSVFSNKAINLKSVASFNTIKTLRFNPPSSPRSLSRNFCRVQATILQDEEEKVVVEESSPLKNFPNDDEPPETGSASALEKWVIKFEQSVNILLTESVIMVLDALYRDRDYARFFVLETIARVPYFAFISVLHMYESFGWWRRADYLKVHFAESWNEMHHLLIMEELGGNAWWFDRFLAQHIAVAYYFVTVFMYVISPRMAYHFSECVESHAFETYDKFIKAQGEKLKKMPAPAVAIKYYTGGDLYLFDEFQTARLPNSRRPKIGKERVLFLALPIRSYSSSIQQY

*>CsAOXb*

MWQAAGANYSSSSLTSGRHLMSRYPAGIVRYWSSASSSSSSSSSSSSAPPVDLPKDKEEINQQDIVSYWGIIPTKVTKEDGSAWRWNCFRDHKPENYRDKFAYWTVQALKFPTHLFFQRRHMCHAMLLQTVAAVPGMVGGMLLHCKSLRKFEHSGGWIKALLEEAENERMHLMTFIELARPQWYERALVFAVQGVFFNAYFLAYLASPKLAHRIVGYLEEEAVNSYTEFLKDLENGSFENAPAPAIAIDYWRMPPDSTLRDVVVDIQCQGHELKDAPAPVGYH

*>CsAOXc*

MYRGGVRLFSTLTARTASTEAAARILAGQTNRHLSTFLVRAPILGSRNRSTLALGGEKEQQREENVQTTGAAAAGGSGNKDEKRIVSYWGVEAPKVNKDDGSEWKWNCFRAWEAYEADLSIDLKKHHAPTTFSDKMALWTVKSLRWPTDLFFQGRYGCRAMMLETVAAVPGMVGGMLLHCKSLRRFEHSGGWIKALLEEAENERMHLMTFMEVAKPKWYERALVFAVQGVFFNAYFLGYLISPKFAHRMVGYLEEEAIHSYTEFLKELDKGNIENVPAPAIATDYWRLPPNSTLKDVVLVVRADEAHHRDVNHYASDIHYQGRQLRESPAPLGYH

*>CsAOXd*

MSVMRGLINGRKHSIGYARTVVRCHPNVWDGDDMPLLGLRMMVMMSSYSSSSESVPEKVKEKGENGIVPSSYWGISRPKITREDGSPWPWNCFMAWETYRADLSIDLKKHHVPTTFLDKVAYRTVKLLRIPTDLFFQGRYGCRAMMLETVAAVPGMVGGMLLHLKSLRKFQHSGGWIKALLEEAENERMHLMTMVELVKPKWYERMLVLTVQGVFFNAFFVLYLLSPKLAHRVVGYLEEEAIHSYTEYLKDIDSGSIENVPAPAIAIDYWRLPKDATLKDVITVIRADEAHHRDVNHFASDIQFQGKELRDAPAPLGYH
