# Supplementary material for: Alternative oxidase (AOX) constitutes a small family of proteins in Citrus clementina and Citrus sinensis L. Osb
Source: PLoS One. 2017 May 1;12(5):e0176878. doi: 10.1371/journal.pone.0176878 (PMC5411082; doi:10.1371/journal.pone.0176878)

**S5 Figure.** **Amino acid sequence identity of CcAOXs and CsAOXs.** The number in each box represents the percentage of identity between AOX proteins. (*) indicates the protein resulting from the alternative transcripts of the gene *CsAOXa*.


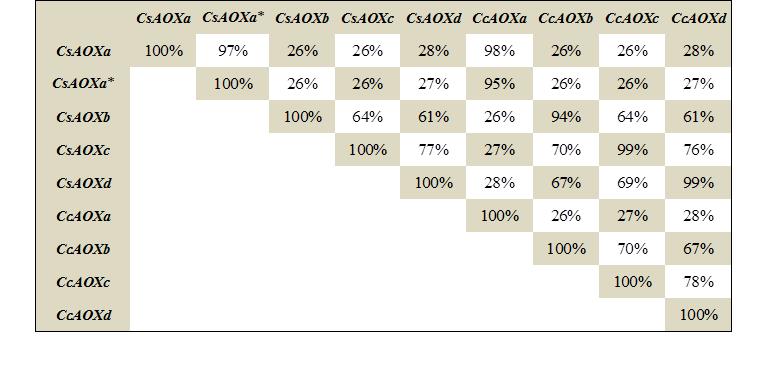

Supplement: S5 Fig — (DOCX) [file pone.0176878.s005.docx]
