# Supplementary material for: Alternative oxidase (AOX) constitutes a small family of proteins in Citrus clementina and Citrus sinensis L. Osb
Source: PLoS One. 2017 May 1;12(5):e0176878. doi: 10.1371/journal.pone.0176878 (PMC5411082; doi:10.1371/journal.pone.0176878)

**S6 Figure. Modeling validation of the CcAOX structure using the Ramachandran plot and the ANOLEA analysis.** Anolea graphic represents energy values for each amino acid of the CcAOX protein. The green bars represent regions of low energy.

**
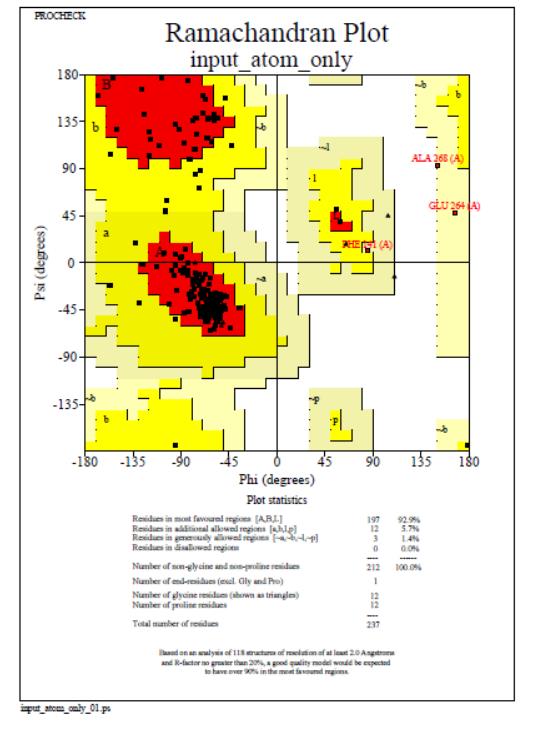
**

**ANOLEA validation**


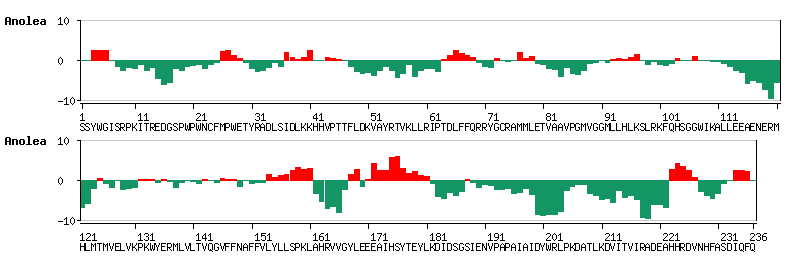

Supplement: S6 Fig — (DOCX) [file pone.0176878.s006.docx]
