# Supplementary figures and images for: Alternative oxidase (AOX) constitutes a small family of proteins in Citrus clementina and Citrus sinensis L. Osb
Source: PLoS One. 2017 May 1;12(5):e0176878. doi: 10.1371/journal.pone.0176878 (PMC5411082; doi:10.1371/journal.pone.0176878)

**S7 Figure. 2-D map of the interaction between CcAOXd and UQ.**

**
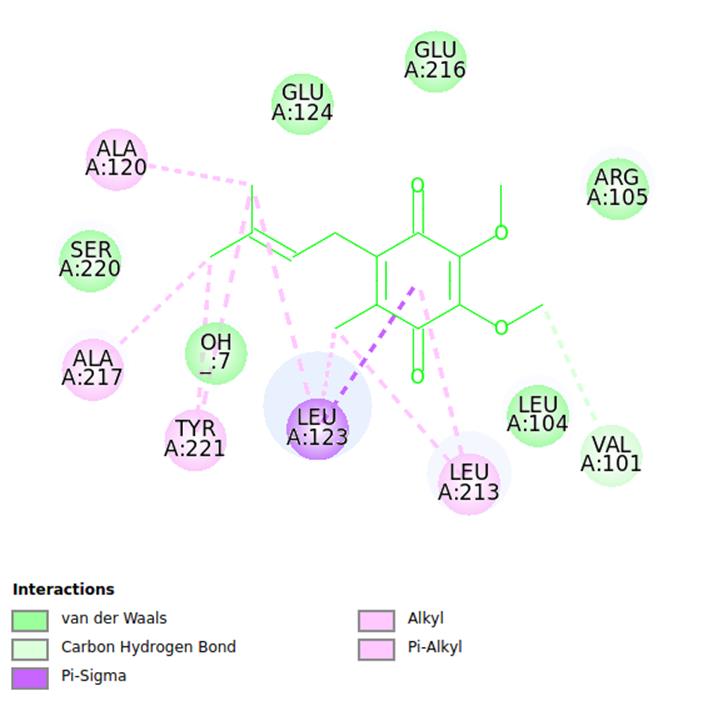
**

Supplement: S7 Fig — (DOCX) [file pone.0176878.s007.docx]
